# Gene Correction Reverses Ciliopathy and Photoreceptor Loss in iPSC-Derived Retinal Organoids from Retinitis Pigmentosa Patients

**DOI:** 10.1016/j.stemcr.2018.02.003

**Published:** 2018-03-08

**Authors:** Wen-Li Deng, Mei-Ling Gao, Xin-Lan Lei, Ji-Neng Lv, Huan Zhao, Kai-Wen He, Xi-Xi Xia, Ling-Yun Li, Yu-Chen Chen, Yan-Ping Li, Deng Pan, Tian Xue, Zi-Bing Jin

**Affiliations:** 1 Lab for Stem Cell & Retinal Regeneration, Institute of Stem Cell Research, Division of Ophthalmic Genetics, The Eye Hospital, Wenzhou Medical University, State Key Laboratory of Ophthalmology, Optometry and Visual Science, Wenzhou 325027, China; 2 Hefei National Laboratory for Physical Sciences at Microscale, CAS Key Laboratory of Brain Function and Disease, Neurodegenerative Disorder Research Center, School of Life Sciences, University of Science and Technology of China, Hefei 230026, China

**Keywords:** RPGR, photoreceptor, electrophysiology, retinitis pigmentosa, patient-derived iPSCs, retinal organoid, RPE cells, cilium, ciliopathy, disease modeling

## Abstract

Retinitis pigmentosa (RP) is an irreversible, inherited retinopathy in which early-onset nyctalopia is observed. Despite the genetic heterogeneity of RP, RPGR mutations are the most common causes of this disease. Here, we generated induced pluripotent stem cells (iPSCs) from three RP patients with different frameshift mutations in the *RPGR* gene, which were then differentiated into retinal pigment epithelium (RPE) cells and well-structured retinal organoids possessing electrophysiological properties. We observed significant defects in photoreceptor in terms of morphology, localization, transcriptional profiling, and electrophysiological activity. Furthermore, shorted cilium was found in patient iPSCs, RPE cells, and three-dimensional retinal organoids. CRISPR-Cas9-mediated correction of RPGR mutation rescued photoreceptor structure and electrophysiological property, reversed the observed ciliopathy, and restored gene expression to a level in accordance with that in the control using transcriptome-based analysis. This study recapitulated the pathogenesis of RPGR using patient-specific organoids and achieved targeted gene therapy of *RPGR* mutations in a dish as proof-of-concept evidence.

## Highlights

• HiPSC-derived 3D retinae with outer segments and electrophysiological properties
• *RPGR* mutation results in diseased photoreceptor in patient iPSC-derived 3D retinae
• Mutation correction rescues defects in photoreceptor morphology and electrophysiology
• Ciliogenesis defects appear in RPGR patient-specific iPSCs, iPSC-RPE, and 3D retinae

## Graphical Abstract

**Figure:**
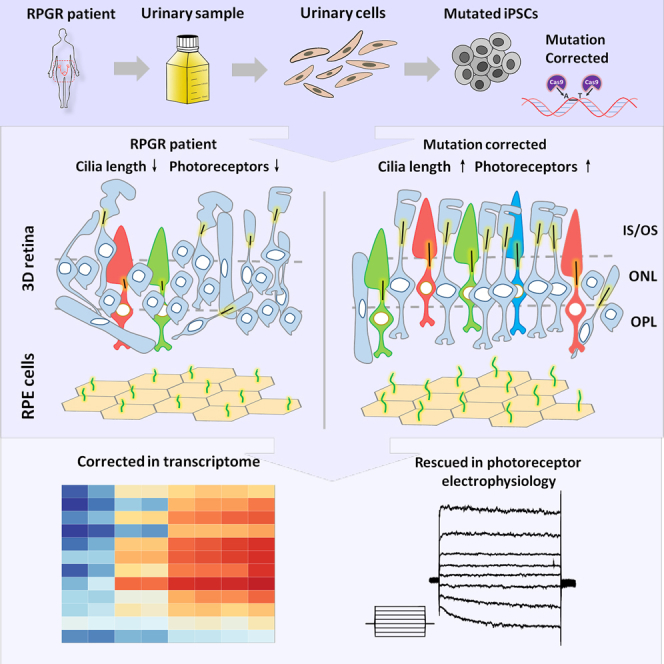

## Teaser

Jin and colleagues demonstrate that patient-specific iPSC-derived 3D retinae can recapitulate disease progress of retinitis pigmentosa through presenting defects in photoreceptor morphology, gene profile, and electrophysiology, as well as the defective ciliogenesis in iPSCs, iPSC-RPE, and 3D retinae. CRISPR/Cas9-mediated gene correction can rescue not only photoreceptor structure and electrophysiological property but also observed ciliopathy.

## Introduction

Retinitis pigmentosa (RP) is a leading cause of blindness worldwide. Unfortunately, this disease is still incurable due to its extreme heterogeneity and unclear mechanisms. To date, more than 85 genes involved in RP have been identified (Daiger et al., 2013, Ran et al., 2014, Huang et al., 2015a). Among these genes, the *RPGR* gene, which was discovered two decades ago (Meindl et al., 1996, Roepman et al., 1996), is one of the most prevalent causative genes, accounting for approximately 16% of RP patients (Vervoort et al., 2000, Hartong et al., 2006, Jin et al., 2006a, Huang et al., 2015b).

The *RPGR* gene is located in the X chromosome, containing 19 exons and one open reading frame (ORF15) (Meindl et al., 1996, Vervoort et al., 2000). The *RPGR* gene has at least two isoforms, RPGR-default and RPGR-ORF15, which share the first 14 exons encoding regulator of chromatin condensation (RCC1) (Meindl et al., 1996, Jin et al., 2006b). RPGR is known as an important component in the centrosome-cilium interface (Gupta et al., 2015). In photoreceptor, it is located in the connecting cilium and *RPGR* mutations can cause cone-rod dystrophy (Hong et al., 2000, Moore et al., 2006). The ORF15 exon is specifically expressed in photoreceptors and contains a substrate of glutamylation; this post-translational modification is critical for its function in photoreceptors (Sun et al., 2016). A large percentage of RPGR mutations causing retinal disease are found to disrupt the ORF15 isoform (Sharon et al., 2003, Megaw et al., 2015). However, the function of ORF15 consisting of glutamic acid/glycine-rich domain is unknown.

Animal models have typically been used to dissect disease mechanisms. The first *RPGR* knockout mouse strain was generated in 2000 (Hong et al., 2000). Cone photoreceptors in these mice are mislocalized and degenerate progressively at a very late age, which is inconsistent with rapid disease progression in RP patients with *RPGR* mutations. The same *RPGR* mutation in two mouse strains with different genetic backgrounds exhibits striking differences in retinal function (Brunner et al., 2010). In canids, different mutations in ORF15 result in truncated RPGR proteins and show marked differences in retinal development and photoreceptor morphology (Zhang et al., 2002). Arduous efforts have been made to elucidate disease mechanisms caused by *RPGR* mutations using animal models. However, there are vast differences in *RPGR* sequences in different species. Thus, it remains challenging to decipher the mechanism of RPGR mutation because of the lack of appropriate study models.

To overcome the roadblocks hampering both mechanistic dissection and drug discovery, substitution of patient-specific diseased retina without ethical restrictions is desired. Induced pluripotent stem cells (iPSCs) generated from terminal somatic cells have greatly facilitated the indirect obtention of diseased cells *in vitro* (Takahashi et al., 2007, Inoue et al., 2014). Using the iPSC approach, we have successfully generated RP-patient-specific rod models that partly recapitulate the disease manifestation (Jin et al., 2011). However, previous methods for retinal differentiation based on two-dimensional (2D) cell culture were unable to generate all structural components, such as the inner and outer segments, or the spatial information for photoreceptor cells, making it difficult to fully recapitulate the disease in a dish (Ikeda et al., 2005, Osakada et al., 2009a). Recently, significant progress has been made in achieving three-dimensional (3D) retinal differentiation from pluripotent stem cells. Eye cups and organic retinae can be produced from both the ESCs and iPSCs via a stepwise method (Eiraku et al., 2011, Nakano et al., 2012, Zhong et al., 2014), which opens an avenue for realizing high-fidelity generation of a patient-specific retina organ *in vitro*.

In the present study, we generate iPSCs from three patients with frameshift mutation in the *RPGR* gene and differentiate these cells into retinal pigment epithelium (RPE) cells and 3D retinae to recapitulate the disease *in vitro*. Using this cutting-edge approach, we were able to demonstrate significant defects in photoreceptors and cilia.

## Results

### Generation and Characterization of RPGR Patient-Derived iPSCs

Urinary cells were isolated from 100–300 mL of urine from three male patients with childhood night blindness who were subsequently diagnosed with RP (Figure S1). Patient 1 harbored a mutation in exon 14 of *RPGR* gene with c.1685_1686delAT, while patients 2 and 3 had mutations in ORF15 of *RPGR* gene with c.2234_2235delGA and c.2403_2404delAG. Urinary cells were reprogrammed into iPSCs with lentivirus for patient 1 or plasmids via electroporation for patient 2 and patient 3 (Figure S2A and Table S1). Control 1, control 2, and control 3 iPSCs were generated from fibroblasts or urinary cells from three healthy volunteers by expression of reprogramming plasmids (Figure S2A and Table S1). At least two independent clonal lines were isolated and expanded in the TeSR-E8 culture system. Three *RPGR* gene mutations were then re-confirmed in the established iPSCs (Figure S2B). The pluripotency of patient and control iPSCs were validated via serial marker staining (Figure S2C). Additionally, the ability to form the three germ layers was verified via *in vitro* differentiation and immunostaining (Figure S2D).

### Structured 3D Retinae with Electrophysiological Property Differentiated from iPSCs

To create a disease model in a dish, iPSCs of patient 1, patient 2, control 1, and control 2 were subjected to 3D retina differentiation following a sophisticated method previously described (Kuwahara et al., 2015) (Figure S3A). Self-forming organoids were induced by timed BMP4 treatment in suspended culture. The ganglion cell marker Brn3b was expressed in the inner layer within both patient and control retinae at week 4 (Figure S3B). At week 6, we did not observe significant differences in differentiation efficiency or organoid morphology between the patient iPSC-derived retinae and control iPSC-derived retinae (Figure S3C). At week 11, the thickness of the neural retinal layer was also similar (Figure S3D). A similar expression pattern of transcription factors that regulate neural retinal development was identified via RNA sequencing (RNA-seq), including eye field transcription factors PAX6, SIX3, RAX, and LHX2, transcription factor CHX10 (which forces retinal progenitor cells to differentiate), and photoreceptor development factor OTX2 (Figures S3E and S3F). Not surprisingly, there were no clear differences between the two independent patient iPSC lines regarding variant retinal cell types and their distribution in the retinal organoids at early stage. These results clearly demonstrated that early-stage retinal organoids could be induced from the patient-specific iPSCs as effectively as from the normal iPSCs.

**Figure 1:**
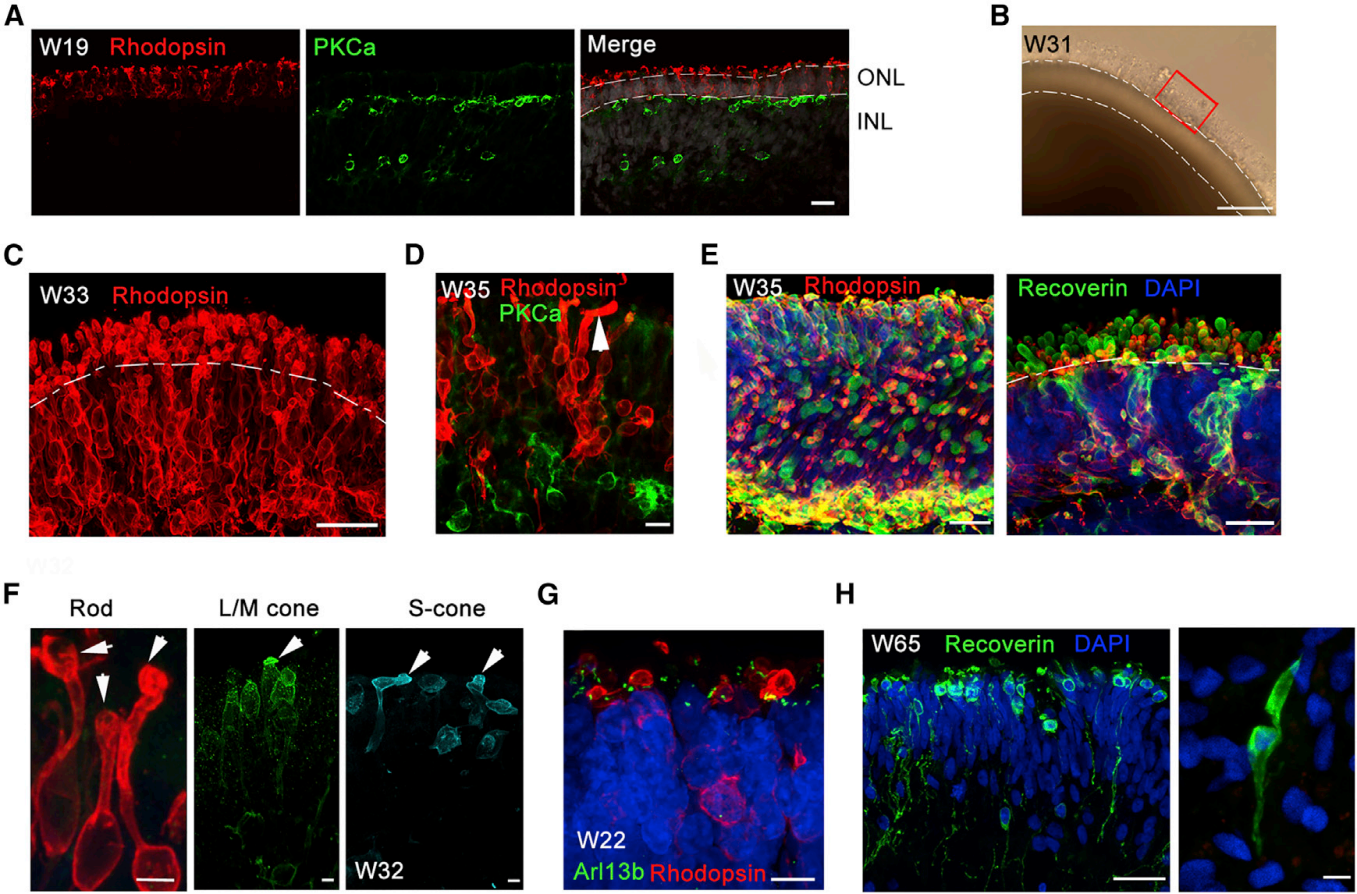
Generation of 3D Retinal Organoids Derived from Control iPSCs with OS-like Structure (A) A polarized structure was formed in which Rhodopsin-positive cells represented the outer nuclear layer, and PKCα-positive cells represented an inner nuclear layer at W19 of the control optic cups. Rhodopsin and PKCα are rod and rod bipolar cell markers, respectively. Scale bar, 20 μm. (B) Bright-field image of a 3D retinal organoid at W31. A red rectangle indicates the OS-like structure on the apical side of the organoid. Scale bar, 100 μm. (C) Rhodopsin-expressing rod cells align on the apical side with a lined OS. Vibratome section. Scale bar, 25 μm. (D) Extended OS of rod photoreceptor (arrow) demonstrates the maturation of photoreceptor cells. Scale bar, 10 μm. (E) Immunostaining of Rhodopsin and Recoverin illustrates the structure of photoreceptor cells in a vibratome section of the control optic cups at W35. Scale bar, 25 μm. (F) Amplified photoreceptor cells expressing rod (Rhodopsin) and cone (L/M-opsin, S-opsin) cell markers. Arrows, OSs. Scale bar, 5 μm. (G) The cilium marker Arl13b reveals the location of connecting cilia, with Rhodopsin^+^ rod cells. Scale bar, 7.5 μm. (H) Long-term culture (W65) of 3D retinae promotes the extension of photoreceptor/cone bipolar axons (left) and cone photoreceptor OS (right). Scale bar, 25 μm (left) and 5 μm (right).

RPGR has been known to localize to the connecting cilium of photoreceptors. RPGR plays a critical role in protein transportation from the inner segment (IS) to the outer segment (OS) (Wang and Deretic, 2014). To study the effect of the *RPGR* mutation on retinal development, and especially photoreceptor genesis, 3D retinal organoids were subjected to long-term differentiation to obtain well-structured photoreceptors containing OS (Figure 1). At week 19, Rhodopsin-expressing rod photoreceptors were aligned in the outer layer adjacent to PKCα-expressing bipolar cells (Figure 1A). The spatial location of rod and bipolar cells was very similar to that in fetal human retina. As the organoid developed, an obvious OS structure was observed (Figure 1B). At week 33, the expression of Rhodopsin was increased with OS located at the apical side of the organoids (Figure 1C). The structure of OS was well preserved using vibratome section (Movie S1). The special location of rods and cones was varied across the organoid; however, rods were predominant at most of the organoid (Movie S2). Moreover, elongated OSs were rarely observed (Figure 1D), and the photoreceptors in the organoid matured further at week 35.

To further define the structure of OS, rods and cones were labeled with Recoverin and their OSs were spherical and raised at the apical side of the organoid. Double staining of Recoverin and Rhodopsin further confirmed the cell types (Figure 1E). Dissected rods and cones detailed the morphology of differentiated photoreceptors bearing OS-like structures (Figure 1F). Similar features were found in fetal retina (Hendrickson et al., 2008). Moreover, the cilium-specific protein Arl13b was expressed, supporting the formation of connecting cilium (Figure 1G). Long-term organoid culture (65 weeks) further promotes the extension of axons of photoreceptor cells and rod bipolar cells. The shape of cone OS is properly formed, indicating the maturation of photoreceptors cells morphologically (Figure 1H). Together, these results indicated the generation of well-structured photoreceptors from iPSCs.

**Figure 2:**
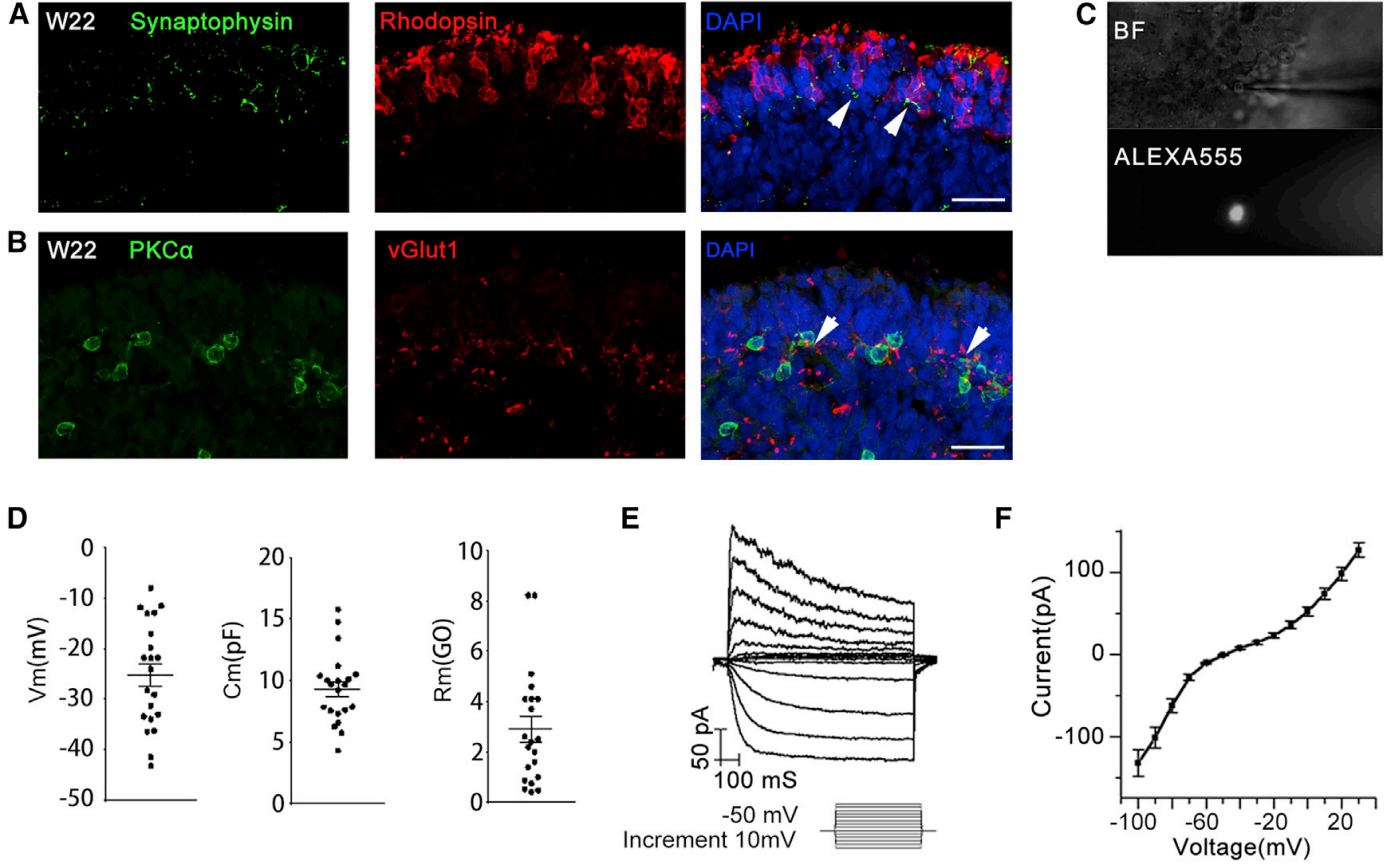
Mature 3D Retinal Organoids Derived from Control iPSCs with Synaptic Connection and Electrophysiological Properties (A and B) Synaptic-specific protein, Synaptophysin, and vGlut1 expression indicates the formation of a connection between rod photoreceptor cells (A, arrows indicate the overlap of Rhodopsin and Synaptophysin) and bipolar cells (B, arrows indicate the overlap of PKCα and vGlut1). Scale bar, 25 μm. (C–F) Examination of the electrophysiological properties of the OS-containing photoreceptor cells in 3D retinal organoid at W24. (C) Representative bright-field (BF) and fluorescent (filled with Alexa 555) images of a recorded cell located at the outer layer of the control iPSC-derived 3D retinal organoid. (D) Resting membrane potential (Vm, left, without junction potential correction), membrane capacitance (Cm, middle), and membrane resistance (Rm, right) of recorded cells. Each black dot represents an individual cell (n = 21). (E) Representative trace of typical current responses of OS-containing photoreceptor cell elicited by a series of voltage steps from −100 mV to 30 mV with a 10 mV increment. (F) Average I-V curve obtained from control iPSC-derived OS-containing photoreceptor cells (n = 21). Results are pooled from three independent experiments. Data are presented as mean value ±SEM.

Furthermore, the expression of synaptophysin and vGlut1 further demonstrated the formation of a connection between photoreceptors (Rhodopsin labeled rods) and the secondary neurons (PKCα labeled bipolar cells) (Figures 2A and 2B), indicating the formation of synaptic connections. Then we also examined the electrophysiological properties of the OS-containing photoreceptor cells (W24) located at the outer layer of the iPSC-derived 3D retinal organoids (Figures 2C–2F). Only relatively small cells with a round OS-like structure were chosen for the experiment (Figure 2C). A total of 21 cells were recorded, with an average membrane capacitance of 9.33 ± 0.63 pF. The average resting membrane potential was −25.22 ± 2.29 mV, and the average membrane resistance was 2.90 ± 0.50 GΩ (Figure 2D). Both were highly consistent with values reported for rods in prior studies under similar recording conditions (Cangiano et al., 2012). Current-voltage relations were also measured under whole-cell configuration using voltage steps from −110 mV to 30 mV with a 10 mV increment (Figure 2E). All recording cells exhibited detectable hyperpolarization-activated current (I_h_) with an I-V curve resembling that of rods (Homma et al., 2013) (Figure 2F). In summary, the rod photoreceptor cells in iPSC-derived 3D organoids possessed electrophysiological property.

### Diseased Photoreceptors in the Patient-Specific Retinae

**Figure 3:**
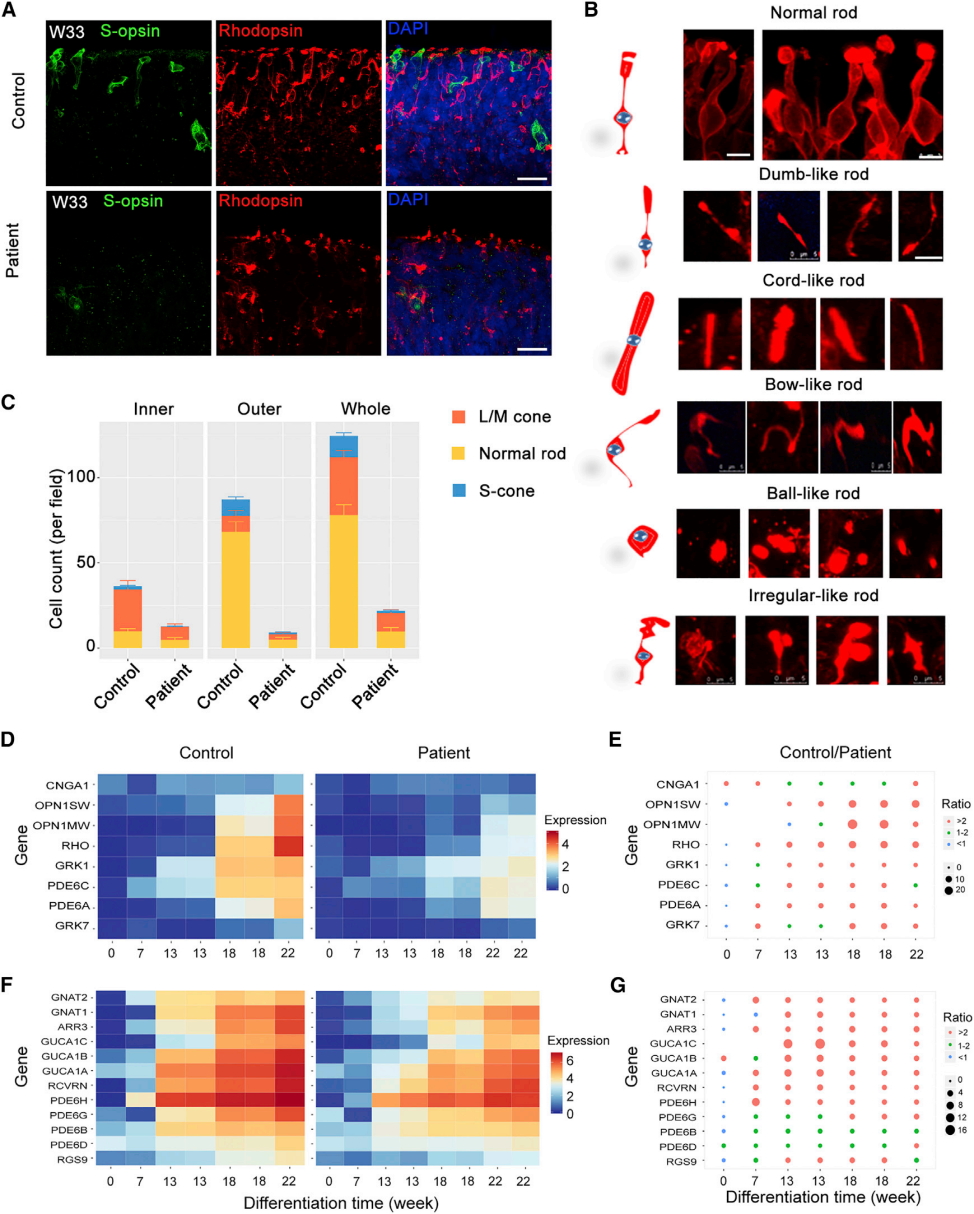
Patient 3D Retinal Organoids Show Diseased Photoreceptors (A) Immunostaining of the rod marker Rhodopsin (red) and the S-cone marker S-opsin (green) shows significant differences in cell morphology and cell counts in retinal organoids at W33. The same phenomenon has been observed in the L/M-cone (see also Figures S3G and S3I). Scale bar, 25 μm. (B) Photoreceptor abnormalities can be observed in patient iPSC-derived 3D retina. Five subtypes of abnormal rod photoreceptors are identified based on the morphology. Scale bar, 5 μm. (C) Comparison of photoreceptor counts in inner, outer, and whole-patient and control retinal organoids. Orange, yellow, and blue represent L/M-cones, normal rods, S-cones respectively. n = 3 organoids for each cell type. Data are from three independent experiments. (D and F) Heatmaps illustrate the gene expression profile of retina-related genes at different organoid stages containing RNA-seq dataset at week 0, 7, 13, 18, and 22. Most of the genes exhibited elevated expression in control retinae compared with that of the patient from week 7. Different colors represent the value of In (FPKM [fragments per kilobase of transcript per million mapped reads] + 1). (E and G) The size of the dot represents the FPKM ratio of control to patient. The genes correspond with those in D and F. Significant differences can be observed in photoreceptor-associated genes. Orange dots, >2 fold change; green dots, 1–2 fold change; blue dots, <1 fold change.

Extremely thin ISs and OSs of photoreceptor layers were observed in patient 1 retinae of both eyes by ophthalmological examinations (Figure S1E). To decipher the effect of the RPGR mutation on photoreceptor development, we further assessed the morphology and cell counts of the differentiated photoreceptor cells in patient 1 and control 1 iPSC-derived 3D organoids. Both rod and cone photoreceptors showed a significantly abnormal morphology, and dislocation of opsins was observed in the patient retinae compared with the control retinae at the corresponding stage (Figures 3A and S3G). The OS structure was clearly shorter in patient retinae than in the control retinae at W27 (Figure S3H).

The expression of Rhodopsin was less affected by *RPGR* mutation than morphology (Figure 3A). To test the hypothesis that RPGR mutation affected Rhodopsin transfer from IS to OS, we next classified the morphologically pathological rods into five obligatory types: bow-like, dumbbell-like, cord-like, ball-like, and irregular rods (Figure 3B). In the patient retinae, the number of each type of defective rods was significantly higher (Figure S3I), while the number of normally shaped photoreceptors was much lower in comparison with control retinae (Figure 3C). These results indicated that more severe impairment of Rhodopsin transport than Rhodopsin expression was induced by RPGR truncation.

Next, RNA-seq analyses were performed based on variant stages of retinal organoids derived from patient and control iPSCs. We then analyzed the expression of genes related to photoreceptor maturation. We found that the expression levels of key genes that regulate photoreceptor maturation were much lower in the patient retinae than in control retinae (Figures 3D and 3E). Notably, the expression of all three genes (OPN1SW, OPN1MW, and Rhodopsin) of the G-protein-coupled receptor 1 family, opsin subfamily encoding S-opsin, L/M-opsin, and Rhodopsin were greatly lower in patient retinae at weeks 13, 18, and 22. CNGA1 encoding a key part of the cyclic guanosine monophosphate (cGMP)-gated cation channel, which allows depolarization of rod photoreceptors, also exhibited a decline in expression in the patient retinae. In control retinae, elevated expression of GRK7, which phosphorylates cone opsins, was found (Figures 3D and 3E). In addition, the expression levels of genes encoding phototransduction enzymes, such as *GUTA1A*, *PDE6A*, and *PDE6C*, showed a remarkable decrease in the patient retinae at all tested time points (Figures 3F and 3G). The decrease of key elements in phototransduction implicated defects of photoreceptor function in the patient retinae. Taken together, these results demonstrated developmental defects in cell morphology and function in photoreceptors in patient-specific retinae.

### Gene Correction Remedies Photoreceptor Development

We repaired the RPGR mutation in patient 1 iPSCs using CRISPR/Cas9 gene editing. Single-guide RNAs (sgRNAs) were designed to target exon 14 near the mutation site and exon 14 was replaced with normal template (Figure S4A). Two mutation-corrected iPSC clones were confirmed via PCR-sequencing (Figures S4B and S4C). The pluripotency of the corrected iPSCs was verified through alkaline phosphatase staining, immunostaining, and *in vitro* differentiation (Figures S4D–S4G).

**Figure 4:**
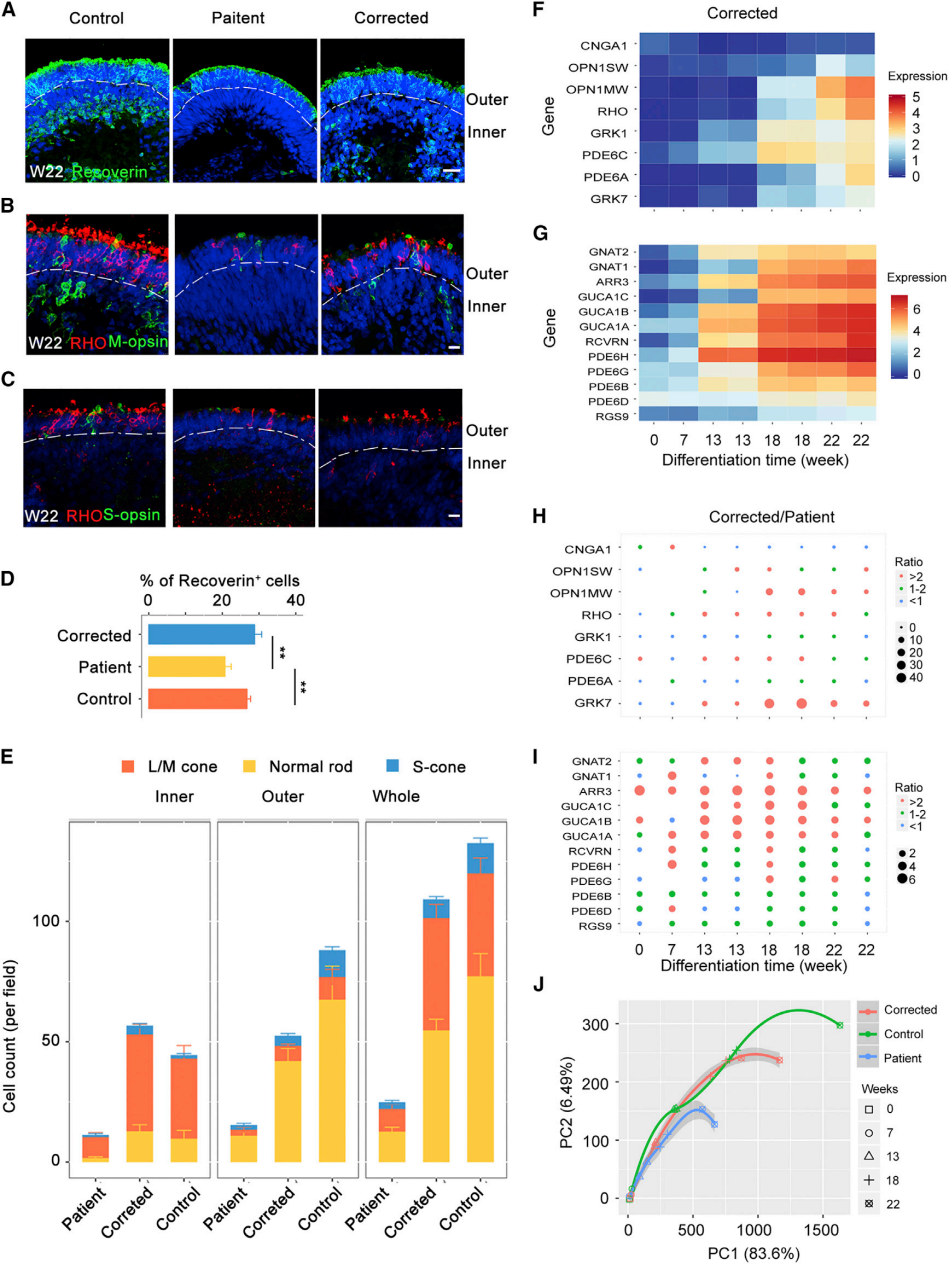
*RPGR* Gene Correction Restores Photoreceptor Defects (A and D) The number of Recoverin-positive cells is lower in patient 3D retinae than in normal and corrected retinae at W22. Scale bar, 25 μm. n = 3 organoids for each cell type. Data are from three independent experiments. Statistical significance was determined using Student's t test, ^∗∗^p < 0.01. (B and C) The number of L/M-cones, S-cones, and normal rods is greater in corrected and control retinal organoids than in patient ones at W22. Scale bar, 10 μm. (E) Quantification of three types of photoreceptors in inner, outer, and whole retinal organoids. The photoreceptor number in patient 3D retinae is highly escalated by *RPGR* gene correction. n = 3 organoids for each cell type. Data are from three independent experiments. (F and G) Heatmaps illustrate the gene expression profile of retina-related genes in corrected 3D retinae RNA-seq dataset at indicated stages. The expression tendency of those genes is similar to the control's and widely different from the patient's (see also Figures 3D and 3F). (H and I) The size of dot represents the FPKM ratio of corrected to patient. The genes are consistent with those in heatmaps. Significant differences in photoreceptor-associated genes can be observed and similar results are found in Figures 3E and 3G. Orange dots, >2 fold changes; green dots, approximately 1–2 fold change; blue dots, <1 fold change. (J) PCA of these 20 photoreceptor-related genes shows a closer correlation between the control and corrected retinal organoids especially at the late stage of retina differentiation. However, the patient retinae show a serious delay of photoreceptor development compared with the control and corrected ones. PC1, 83.6%; PC2, 6.49%.

To test the commitment to photoreceptor development after mutation repair, the iPSCs were differentiated into 3D retinal organoids as described above, and the structure of the organoids was assessed via immunostaining (Figures S4H–S4K). At week 22, both Recoverin expression and cell morphology were restored in the mutation-corrected patient retinae (Figures 4A–4C). Additionally, the decreased number of rods and cones was reversed via mutation repair (Figures 4D and 4E). Moreover, similar expression patterns of photoreceptor-related genes were revealed in mutation-corrected patient retinae compared with normal retinae through RNA-seq (Figures 4F–4I). A principal component analysis (PCA) based on expression profile of 20 photoreceptor-related genes further revealed a close correlation of the 3D retinal organoids derived from corrected and control iPSCs, rather than the patient iPSCs (Figure 4J). To determine the mechanism that may underlie photoreceptor impairment caused by RPGR mutation, cell apoptosis and cell cycle were analyzed (Figure S5). The expression of necrosis and inflammation receptors, such as TNFRSF10D, TNFRSF10B, FAS, and IL1R1, were all upregulated in patient organoids but reduced to normal level in corrected ones (Figure S5A). However, no obvious differences were found in expression of a typical apoptotic marker, caspase-3 (Table S2 and Figure S5B). After gene correction, the TUNEL-positive cell count was significantly decreased in the inner layer of retinal organoids (Figures S5C and S5D). To find out whether other pathways are involved in photoreceptor death and apoptosis, we analyzed enriched genes in the cell cycle pathway. The expression of CDKN2A, CDC6, PTTG1, CDK1, CCNB1, MAD2L1, and E2F2 was upregulated. In addition, elevated expression of those genes was rescued in corrected ones (Figure S5E). The expression of CDKN2A, a member of the p53 signaling pathway, was increased, which might cause apoptosis or necrosis in patient organoids. These results demonstrated that the developmental defects of the photoreceptors were recovered via CRISPR-Cas9-mediated gene correction in patient retinae. This result implies that correction of the RPGR mutation leads to repair of imperfections in photoreceptor development.

**Figure 5:**
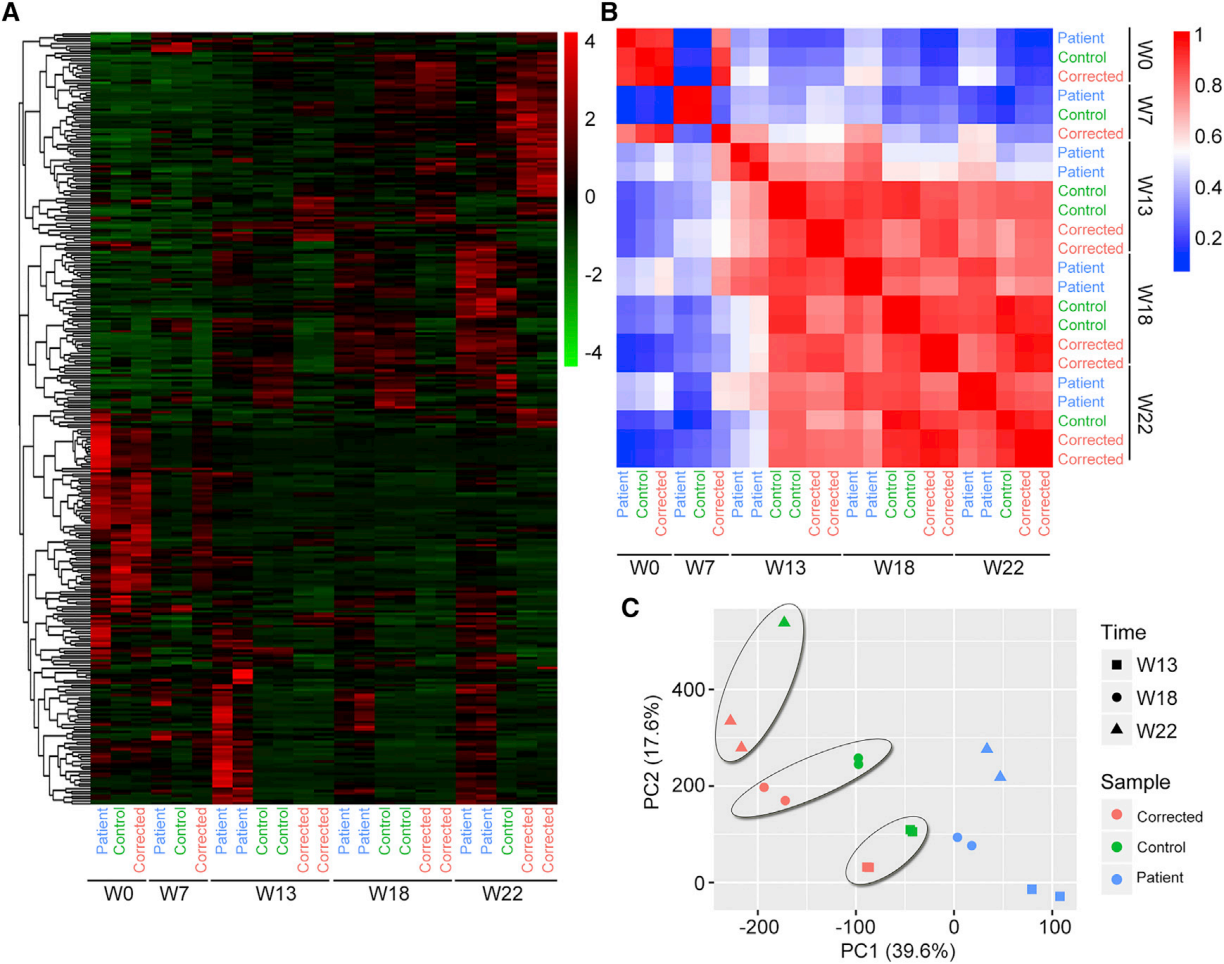
The RPGR Gene Mutation Affects RNA Expression in the Late Stage of Patient Retinal Organoids (A) Hierarchical cluster based on gene expression in each sample. Heatmap shows simplified transcriptome variations between the patient 1, control 1, and corrected 1 retinal organoids at W0, W7, W13, W18, and W22. Two biological replicates were performed for the patient and corrected samples at W13, W18, and W22 and for the control samples at W13 and W18. (B) Heatmap showing Pearson's correlation between patient, control, and corrected retinal organoids at different time points. (C) PCA of 314 genes that significantly changed in control and corrected retinal organoids in comparison with patient ones (fold change >2 or <2) showing a closer correlation between the control and corrected retinal organoids at W13, W18, and W22. PC1, 39.6%; PC2, 17.6%.

To assess differences in transcriptome signatures, we further investigated and compared the expression patterns of retinal-related genes in the 3D retinal organoids derived from patient, control, and corrected iPSCs. First, hierarchical cluster analysis of the week 0 and 7 datasets showed small variation between organoids derived from different iPSC lines (Figure 5A). Significant similarity between the two repeats from the same iPSC line was found at weeks 13, 18, and 22. Furthermore, Pearson's correlation analysis showed that the organoids derived from corrected iPSCs were strongly correlated with those from the control iPSCs, while the correlation with those from patient iPSCs was lower at weeks 13, 18, and 22 (Figure 5B). Additionally, PCA revealed a closer correlation between the corrected and control organoids, in contrast with the patient organoids (Figure 5C). Collectively, these results demonstrated that RPGR correction rescued transcription defects in patient 3D retinal organoids.

In the retina, GFAP was normally found in astrocytes in the ganglion cell layer. However, in RPGR patient retinae, GFAP labeling of Müller cells were activated throughout the thickness of the retina (Hong et al., 2000). We observed the same phenomenon in patient-derived retinal organoids. The expression level of GFAP was more highly upregulated in patient retinal organoids at W75 than in control ones at corresponding stages (Figure S6). After correction of the RPGR mutation, the expression of GFAP protein was reduced to a similar level to control ones (Figure S6C).

**Figure 6:**
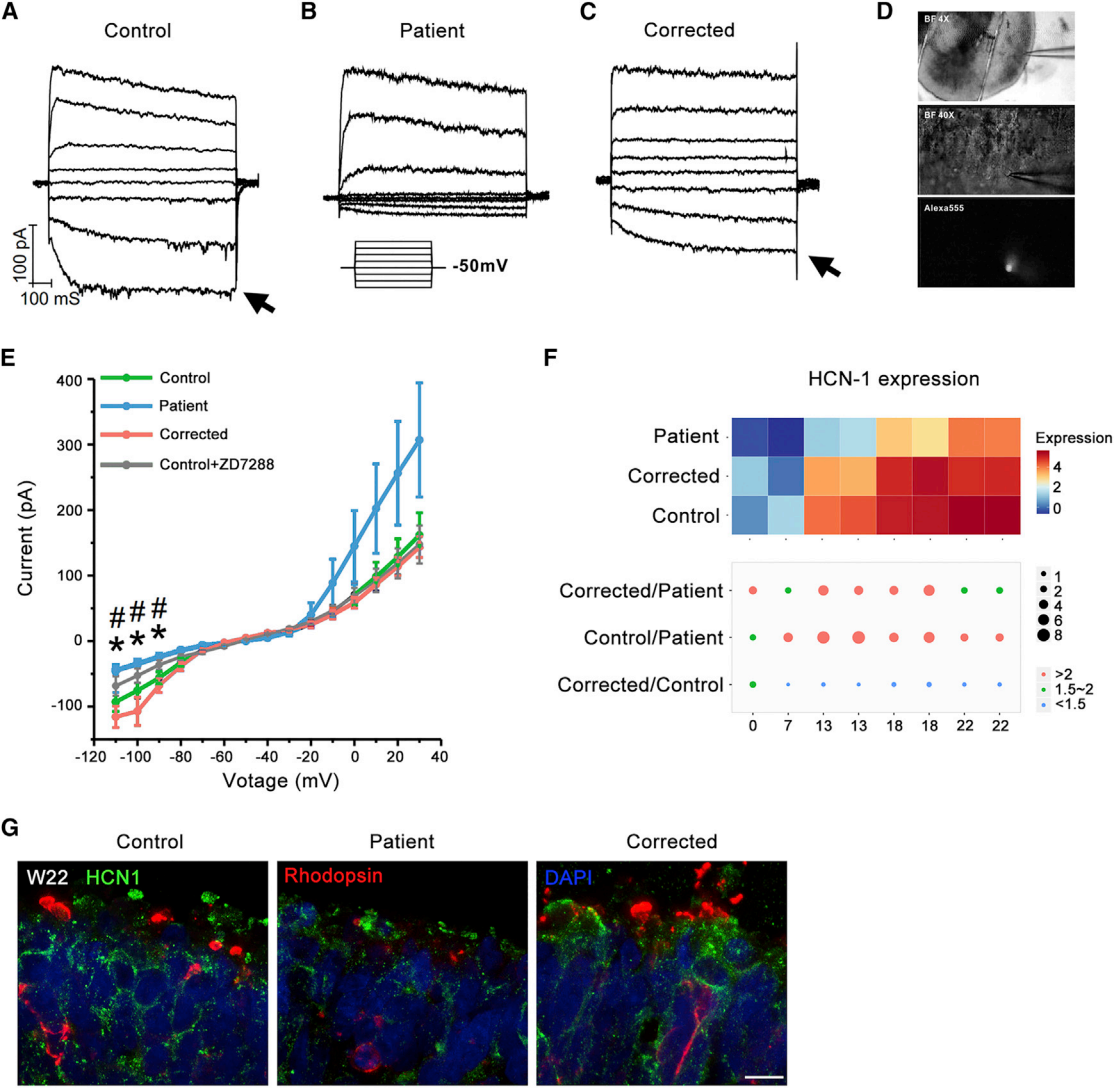
Genetic Correction Restored Hyperpolarization-Activated Potassium Current (I_h_) in Cells from Patient Retinal Organoids at W36 (A–C) Representative traces of current responses elicited by a series of voltage steps from −110 mV to 30 mV with a 20 mV increment (protocol shown as inset). Notice I_h_ (arrow) in cells recorded from control retinal organoids (A), which was absent from patient ones (B) and restored in corrected ones (C). (D) BF and fluorescent (filled with Alexa 555) images of a recorded cell located at the outer layer of retinal organoids. (E) Comparison of I-V curves of cells recorded from control 1 (blue, n = 24), patient 1 (orange-red, n = 21), and corrected 1 retinal organoids (green, n = 15). I_h_, activated between −80 mV and −110 mV, was significantly larger in cells from control and corrected organoids, and markedly reduced by the application of 40 M HCN blocker ZD7288 (wild-type [WT] + ZD7288, gray). Asterisk (^∗^), patient retinal organoids significantly different from control and corrected. Hash (#), significant difference between WT and WT + ZD7288. Results are pooled from three independent experiments. Data are presented as mean ± SEM. Statistical significance was determined using unpaired t test. (F) Heatmaps illustrate the gene expression profile of HCN-1 in RNA-seq dataset at week 22. Different colors represent the value of log2 (FPKM+1). The size of dot represents the FPKM ratio of corrected to patient, control to patient, and corrected to control. Orange dots, >2 fold changes; green dots, approximately 1.5–2 fold change; blue dots, <1.5 fold change. (G) Sample images of the immunostaining of patient, corrected, and control retinal organoids at W22. Scale bar, 7.5 μm.

We further compared the electrophysiological properties of the rod-like cells located at the outer layer of the control, patient, and corrected retinal organoids at W36. Only relatively small cells with a round morphology were chosen for the experiment (Figure 6D). Cells from different groups were comparable with regard to membrane capacitance (control, 8.9 ± 0.5 pF; patient, 8.2 ± 0.5 pF; corrected, 9.1 ± 0.4 pF) and membrane resistance (control, 3.4 ± 0.4 GΩ; patient, 3.5 ± 0.6 GΩ; corrected, 2.3 ± 0.5 MΩ). We then examined the expression of functional HCN (hyperpolarization-activated cyclic nucleotide-gated) channels, which are characteristic of photoreceptors, by measuring hyperpolarization-activated potassium current (I_h_). As expected, cells recorded from control retinal organoids exhibited prominent I_h_ (arrow in Figures 6A and 6E blue) that could be effectively blocked by the HCN inhibitor ZD7288 (Figure 6E gray). On the other hand, significantly less I_h_ was observed in cells from patient retinal organoids (Figure 6B), with a trend of showing greater outwardly rectifying potassium current (Figure 6E orange-red), indicating a more general but not photoreceptor-directed neuronal identity. This deficit, however, was completely reversed by the genetic modification in the corrected retinal organoids (Figures 6C and 6E green), suggesting that genetic correction restored photoreceptor-like properties at the electrophysiological level.

To further confirm the deficit of HCN channels, we analyzed their expression. Based on RNA-seq data, the gene expression of HCN-1 was significantly decreased in patients’ retinae and the deficit was rescued in corrected ones (Figure 6F). In consistent with RNA-seq data, the immunostaining result also demonstrates the decrease of HCN-1 expression, especially in the outer layer of the retina. In the control and corrected retinae, the HCN-1 channels are specifically located in the membrane and ISs of photoreceptor cells (Figure 6G). Collectively, these data suggested that the decreased expression of HCN-1 channels might cause the impairment of I_h_.

### Ciliopathy Is Salvaged through Gene Correction

**Figure 7:**
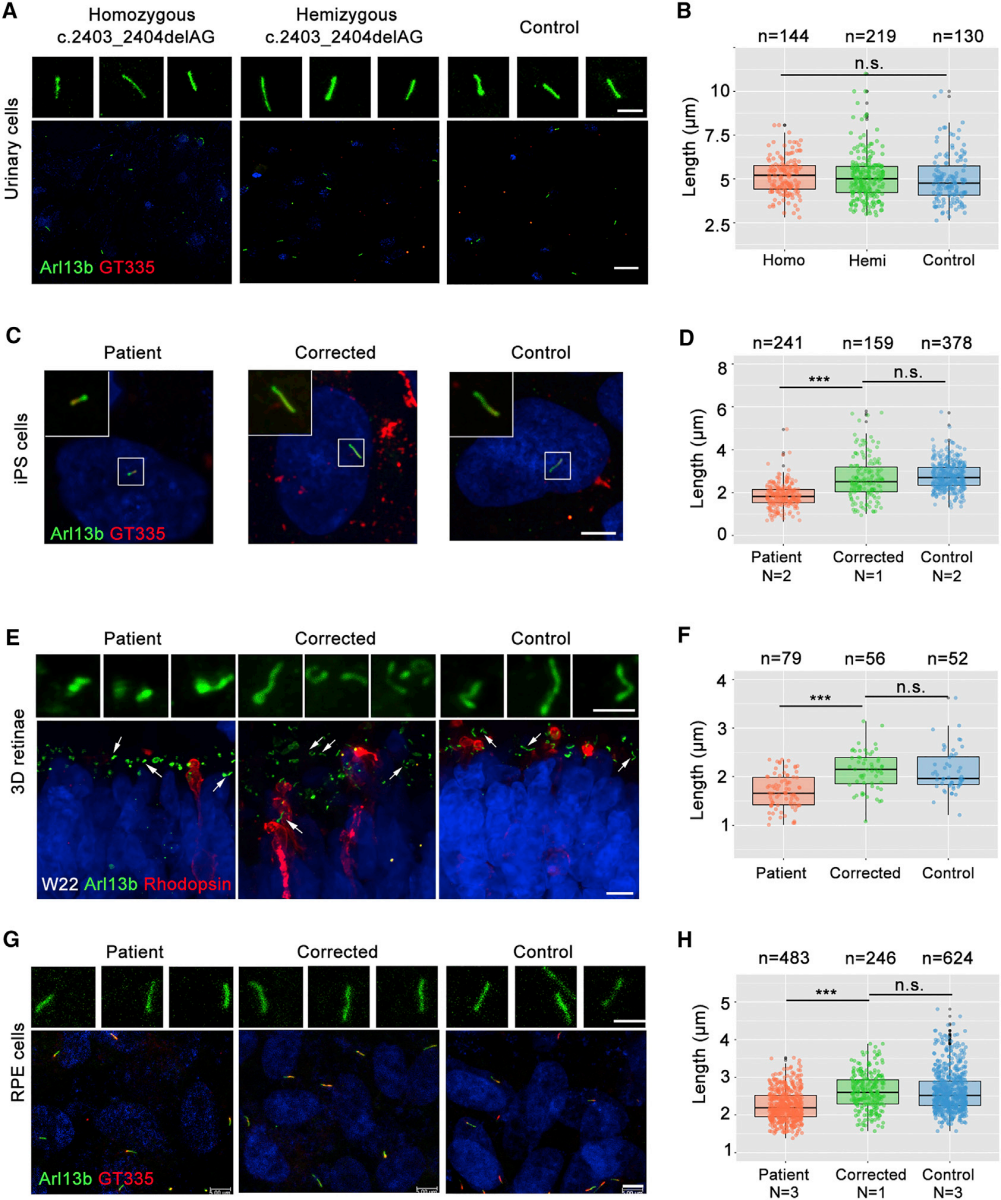
RPGR Gene Correction Rescues Cilium Elongation (A, C, E, and G) Representative immunofluorescence images stained with cilium marker, GT335 (red) and Arl13b (green). (B, D, F, and H) Quantification of cilia length presented in (A), (C), (E), and (G), respectively. All cell numbers counted in each group are obtained from three independent experiments and shown above the graphs. Color dots indicate each cilium, black dots indicate the deviation of the data, and the three horizontal black lines represent the upper quartile, the median, and the lower quartile respectively. ^∗∗∗^p < 0.001, n.s., not significant, unpaired t test. (A) Urinary cells from a nuclear family of RPGR mutation, including homozygote (patient 3), hemizygote (patient 3’s mother), and familial control (patient 3’s father). Scale bar, 5 μm (up) and 25 μm (down). (C) Scale bar, 5 μm. (D) The cilia lengths of iPSCs of control 1, control 2, patient 1, patient 2, and corrected 1 are presented. Donor numbers are indicated with N below the graphs. (E) Arrows indicate the magnified cilia showing above. Scale bar, 2 μm (up) and 7.5 μm (down). (F) n = 3 organoids for each cell type. (G) Scale bar, 2.5 μm (up) and 5 μm (down). (H) The cilia lengths of iPSC-derived RPE of three controls, three patients, and corrected 1 are measured. Donor numbers are indicated with N below the graphs.

RPGR is known to play a critical role in ciliogenesis. Therefore, first we assessed cilia length in serum-starved urinary cells from a male patient (patient 3) with homozygous c.2403_2404delAG RPGR mutation; his mother, who was hemizygous for the mutation; and his father as a normal control (Figure S7A). Primary cilia length of urinary cells was identified by immunostaining of cilium-specific proteins, Arl13B and GT335. The primary cilia lengths in patient (5.177 μm), carrier (5.098 μm), and control (4.992 μm) urinary cells had no significant differences (Figures 7A and 7B). Then, we tested the primary cilia length in iPSCs of patient 1, patient 2, control 1, control 2, and corrected 1. Specifically, the length of the cilia in the patient iPSCs was 1.853 μm, which was significantly shorter than that of control iPSCs (2.774 μm, p < 0.001). In RPGR-corrected patient iPSCs, the length was 2.653 μm, indicating that this ciliopathy was rescued by correction of the RPGR mutation (Figures 7C and 7D).

Next, we investigated the length of the connecting cilia in photoreceptors of 3D retinae differentiated from patient 1 iPSCs. Shortened cilia were again observed in the patient retinae in comparison with those in the control (1.700 vs 2.112 μm), which were mended to normal level (2.125 μm) via mutation repair in the same manner as described above (Figures 7E and 7F). Finally, we wanted to find out whether the cilia length is affected by RPGR mutations in polygon mesh iPSC-derived RPE cells. After differentiation and culture for more than 60 days, polygon mesh iPSC-derived RPE cells containing pigment were used for cilia analysis (Figures S7B and S7C). Cilia in RPE cells derived from patient 1, patient 2, patient 3, corrected 1, control 1, control 2, and control 3 were measured (Figures 7G and 7H). Those cilia were shorter in three patient RPE cells than in control ones (2.255 μm vs 2.633 μm), and gene correction repaired the cilia length to 2.626 μm in RPE cells. Collectively, these findings indicate that ciliation defects in patient iPSCs, shortened cilia in RPE cells, and 3D retinae can be reversed via *RPGR* gene correction, further supporting the existence of ciliopathy in retinae with this mutation.

## Discussion

In this study, we utilized patient-specific iPSCs, RPE cells, and 3D retinal organoids to recapitulate RP predisposed by three different *RPGR* mutations. Through in-dish disease modeling combined with gene correction, we were able to elucidate photoreceptor developmental defects and ciliopathy in patient retinae. We successfully generated iPSCs from three male patients harboring c.1685_1686delAT, c.2234_2235delGA, and c.2403_2404delAG mutations in the *RPGR* gene respectively, and simulated retinal development via the differentiation of retinal organoids. Decreases in retinal gene expression, photoreceptor cell numbers, and cilia length were observed in the patient-derived 3D retinae and RPE cells. Significantly, these defects were restored via *RPGR* gene correction.

The 2 bp deletion in exon 14 and ORF15 in our patients resulted in a pre-matured RPGR protein with complete or partial loss of ORF15 or exons 15–19, which is the genetic predisposition responsible for impaired ciliogenesis (Zhang et al., 2002), which is consistent with our finding that cilia length in patients’ iPSCs, RPE cells, and retinae was much shorter than that of controls (Figure 7). Defects in expression of genes encoding intraflagellar transport proteins were also found, especially in RPGRIP1, which interacts with RPGR directly (data not shown).

*In vitro* differentiation from stem cells to retinal cells made great advancement in recent years. We made the first attempt at disease modeling and drug testing using iPSCs derived from five RP patients (Jin et al., 2011). Precise special orientation and development of OS are critical for visual function, which was unattainable with 2D culture (Ikeda et al., 2005, Osakada et al., 2009a). A new retinal disease model was used until 3D differentiation of human neural retinae succeeded (Nakano et al., 2012, Kuwahara et al., 2015, Zhong et al., 2014). Recently, Leber congenital amaurosis models were created using patient iPSC-derived optic cups originating from fibroblasts (Parfitt et al., 2016, Shimada et al., 2017). Here, we made the first attempt to interrogate RPGR mutation mechanisms using patient-specific retinae. After long-term culture, iPSC-derived 3D retinae exhibit a polarized photoreceptor cell layer containing an OS-like structure and obtaining electrophysiological properties, providing us with patient-specific retinae to recapitulate the pathogenesis caused by the RPGR mutation in the present study (Figure 2). RPGR is known as one of the components in the molecular complex that controls Rhodopsin transport to the OS (Wang and Deretic, 2014). In the patient retinae, delocalized Rhodopsin was observed, indicating impaired passage of proteins (Figures 4A and 4B). Impairments in expression of genes related to visual transduction further demonstrated the malfunction of photoreceptor (Figures 4D and 4E). Additionally, the defect in electrophysiological properties in patient retinae might be caused by impairment of HCN-1 channels (Figure 6) (Schon et al., 2016). Compared with non-human animal models, the patient-specific retinae model recapitulates the disease phenotype better and is therefore useful for studying the mechanisms involved and for drug discovery.

We also observed defective photoreceptor development and ciliopathy in the patient-specific retinae (Figures 7E and 7F). The shorter cilia were also found in retinal organoids derived from patients with CEP290 mutation (Parfitt et al., 2016, Shimada et al., 2017). Both RPGR and CEP290 are key proteins in ciliogenesis. Here, we compared cilia length of urinary cells from a nuclear family with RPGR mutation (c.2403_2404delAG), including a homozygote, a hemizygote, and a familial control. Similar cilia lengths were found in three members of this family (Figures 7A and 7B). However, cilia lengths of fibroblast were affected differently in patients with different CEP290 mutations (Parfitt et al., 2016, Shimada et al., 2017). Primary cilia of RPE cells derived from patient iPSCs exhibited significantly reduced lengths compared with those of control and corrected ones (Figures 7G and 7H), which was consistent with previous reports. They also found the decreased cilia length in RPE cells of Leber congenital amaurosis patients (Parfitt et al., 2016).

Defects were observed in morphology and cell counts of the differentiated photoreceptor cells in patient-derived 3D organoids. To find out the molecular basis of RP caused by RPGR mutation, gene expression profiles were analyzed at different time points of retinal organoid induction. From the analysis of RNA-seq data, no significant differences were found in the expression of genes of typical apoptosis, autophagy, and endoplasmic reticulum (ER) stress (Table S2). However, a few necrosis-related genes, including FAS, TNFRSF10D, TNFRSF10B, and TNFSF10, in apoptotic pathways are significantly upregulated in patient organoids. The recovery of the gene expression is also found in corrected organoids (Figure S5A). No obvious differences of proliferation and apoptosis can be observed in iPSCs from patients and controls (Table S2). In a previous study, increased expression of ER stress and apoptotic markers was found in iPSC-derived photoreceptor cells with Rhodopsin mutation (Yoshida et al., 2014). Thus, we also performed TUNEL and immunostaining of an apoptotic marker, activated caspase-3 (Figure S5). No obvious differences of caspase-3 level have been observed among patient, control, and corrected retinal organoids (Figure S5B). Few TUNEL-positive cells are found in the neural retinal cells layer and the cell count in patient 1 retinal organoid is significantly higher than that in control and corrected retinal organoids (Figures S5C and S5D). In addition, we found significant defects in gene expression in the cell cycle signaling pathway in patient organoids at W13 (Figure S5E). The expression of transcription factors, tumor suppressors, and genes that play essential roles in G1/S and G2/M phase transitions of eukaryotic cell cycle is significantly upregulated in patient organoids (Cheong et al., 2006, Landis et al., 2006). The impaired expression of genes in the cell cycle might be one of the reasons for decreased cell count of photoreceptor cells. Further studies will be needed to address the mechanism of defect in retinal organoid of patients with RPGR mutations.

We successfully recapitulated RP predisposed by the *RPGR* mutation using patient-derived retinae in a dish. The defects in patient iPSC-derived retinae are consistent with the clinical phenotype (Figures 3, S3, and S1). These patient-derived organoids may be useful for drug screening and predicting drug side effects. Our study also demonstrated that diseased photoreceptors can be restored via gene correction, providing *in vitro* proof-of-concept evidence supporting the adopted mutation repair strategy using CRISPR/Cas9-mediated gene editing. Moreover, the mutation-corrected patient retinae are similar to healthy retinae and are potentially of value for retinal regeneration, without any concern regarding immunological rejection (Giacalone et al., 2016). In summary, this study interrogated the disease *in vitro* utilizing RPGR patient-derived 3D retinal organoids. Our findings regarding photoreceptor pathogenesis may suggest treatments for patients.

## Experimental Procedures

### Cell Culture and Generation of iPSCs

We isolated urinary cells from 100–300 mL of urine from three RPGR patients, one familial carrier, and three controls as previously reported (Zhou et al., 2012). Human iPSCs were generated with a lentiviral pVSVG vector (Addgene) containing human Oct4, Sox2, Klf4, and c-Myc cDNAs or a cocktail of reprogramming plasmids encoding Oct4, Sox2, Lin28, Klf4, L-myc, p53shRNA, and the miR-302/367 cluster (Episomal iPSC Kit, System Biosciences) delivered by nucleofection using LONZA 4D. All human subjects were treated according to institutional guidelines approved by the Medical Ethics Committee at the Eye Hospital of Wenzhou Medical University.

### Generation of Retinal Organoid and RPE Cells

3D retinae were generated from iPSCs through a method as previously described (Kuwahara et al., 2015). RPE cells were differentiated following the method previously reported (Osakada et al., 2009b).

### Electrophysiology

A 3D retinal organoid at 24 or 36 weeks was transferred to the recording chamber and gently held down with a platinum weigh crossed with fibers. The chamber was constantly perfused with Ames solution equilibrated with 95% O_2_/5% CO_2_. Only small cells with a round OS-like structure located at the outer layers of the 3D retinal organoid were chosen for patch-clamp recordings; for visualization of the recorded cell, 50 μM of Alexa Fluor 555 dye (Thermo Fisher) was included in the pipette solution. Recording pipettes were pulled with a tip resistance between 4 and 7 MΩ, and backfilled with pipette solutions containing 110 mM KCl, 13 mM NaCl, 2 mM MgCl_2_, 1 mM CaCl_2_, 10 mM EGTA, and 10 mM HEPES (pH 7.2) adjusted with KOH, supplemented with 5 ATP-Mg and 0.5 GTP-Na. Recordings were made with a Multiclamp 700B amplifier and a Digidata 1440A digitizer. Data were acquired with Axon pClamp software (Molecular Devices), low-pass filtered at 1,000 Hz, and sampled at 20 kHz.

### CRISPR/Cas9-Mediated Genome Editing

sgRNAs were designed and constructed into the pX330 plasmid. Homologous recombinant template was amplified from control DNA and cloned into pEASY-Blunt simple cloning vector (TransGen Biotech, Beijing) to construct a 3.4 kb targeting vector carrying a neomycin selection cassette. Both of the constructed vectors were delivered into patient iPSCs by electroporation (LONZA 4D) and then selected with G418. Finally, the mutation-corrected clones were identified by PCR and sequencing after G418 selection.

### Statistics

Statistical significances were analyzed using one-way ANOVA, unpaired t test, Student's t test, and Turkey's post hoc test as indicate in the figure legends. ANOVA was realized with either the nonparametric Friedman test followed by Dunn's multiple comparison test or the Mann-Whitney test for all pairwise analyses (Prism 6; GraphPad software).

## Author Contributions

W.-L.D. and M.-L.G. designed and performed experiments, analyzed data, prepared figures, and wrote the manuscript. X.-L.L., Y.-C.C. and D.P. conducted most of the immunostaining, cilium imaging, and data analysis. J.-N.L. performed gene correction. H.Z., L.-Y.L., and T.X. carried out patch clamping. K.-W.H. and Y.-P.L. conducted immunostaining and analysis. X.-X.X. identified the pluripotency of iPSCs. Z.-B.J. conceived the idea for the project, supervised experiments, analyzed data, wrote the manuscript, and provided funding support.

## Accession Numbers

The accession number for the RNA-seq data reported in this paper is ArrayExpress: E-MTAB-6490.

## Supplemental Information

Document S1. Supplemental Experimental Procedures, Figures S1–S7, and Table S1

Table S2. Compiled Comparison of RNA-Seq Data Related to Figures 3 and 4 (Sheet 1), Figure 5 (Sheet 2), Figure 6 (Sheet 3), Figure S3 (Sheet 4), Figure S5 (Sheet 5), and Other Genes (Sheet 6)

Movie S1. Differentiated Rod (Rhodopsin, Red) and S-Cone (S-Opsin, Green) Showing the OS-like Structure (a Higher-Magnification View), Related to Figure 1

Movie S2. A Lower-Magnification View of a Differentiated 3D Organoid Showing the Distribution of Rods (Rhodopsin, Red) and L/M-Cones (M-Opsin, Green), Related to Figure 1

Document S2. Article plus Supplemental Information
