# Supplementary material for: Gene Correction Reverses Ciliopathy and Photoreceptor Loss in iPSC-Derived Retinal Organoids from Retinitis Pigmentosa Patients
Source: Stem Cell Reports. 2018 Mar 8;10(4):1267–81. doi: 10.1016/j.stemcr.2018.02.003 (PMC5998840; doi:10.1016/j.stemcr.2018.02.003)
Supplement: Document S1. Supplemental Experimental Procedures, Figures S1–S7, and Table S1 [file mmc1.pdf]

Stem Cell Reports, Volume *10*

Supplemental Information

Gene Correction Reverses Ciliopathy and Photoreceptor Loss in iPSC-Derived Retinal Organoids from Retinitis Pigmentosa Patients

Wen-Li Deng, Mei-Ling Gao, Xin-Lan Lei, Ji-Neng Lv, Huan Zhao, Kai-Wen He, Xi-Xi Xia, Ling-Yun Li, Yu-Chen Chen, Yan-Ping Li, Deng Pan, Tian Xue, and Zi-Bing Jin

Supplemental Figures and legends

Figure S1

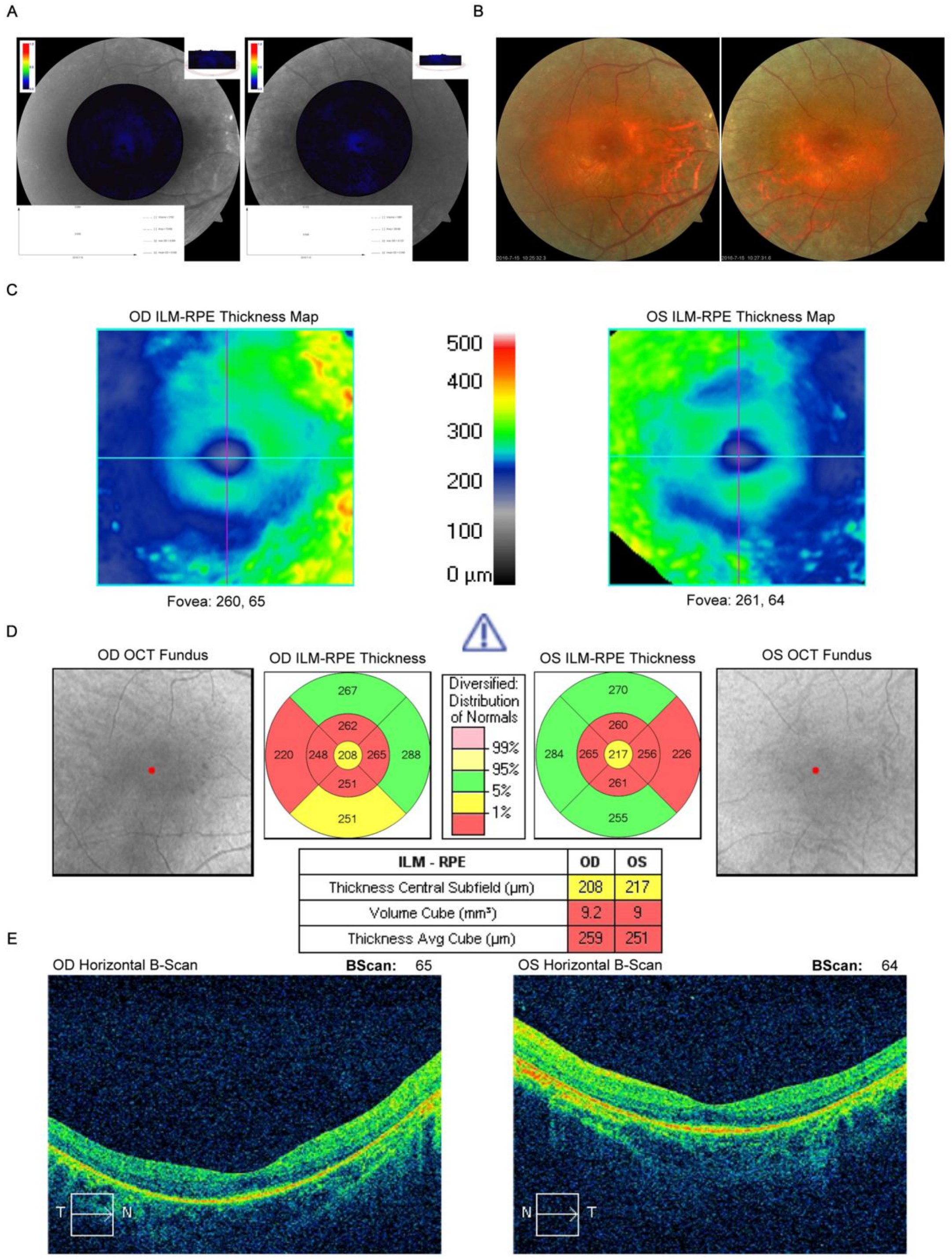

Figure S1. Phenotype information for the RPGR patient-1. Related to Figure 3 and 6. (A) The macular pigment optical density (MPOD) of RPGR patient at 25 years

old, it was captured by one-wavelength fundus reflectance method. Four parameters, including max OD (optical density), mean OD (optical density), volume and area are all remarkably decreased in both eyes. (B) Fundoscopy of patient with RPGR mutation displays typical retinitis pigmentosa features including peripheral bone-spicule pigmentation. At the age of 25. (C) The general impression of the macular cube 512 x 128 ILM-RPE layer heat map. Different colors represent different thickness of the RPGR patient retina. The temporal parts of ILM-RPE layers in both eyes are thinner than 300 μm. (D) Details of the macular cube 512 x 128 ILM-RPE thickness heatmap. Red, diversified distribution of controls is less than 1%, yellow, the distribution ranges from 1% to 5%, green, the distribution is 5% to 95%. The fovea and temporal part of both eyes ILM-RPE thickness declined most obviously. The volume cube and thickness of average cube as well as the thickness of central subfield decreased significantly. (E) Optical Coherence Tomography (OCT) horizontal b-scan displayed extremely thin inner and outer segments of photoreceptor layers in RPGR patient retina of both eyes. Some temporal parts are even absent from photoreceptors which are consistent with the phenomenon found in our patient-specific 3D retinae.

Figure S2

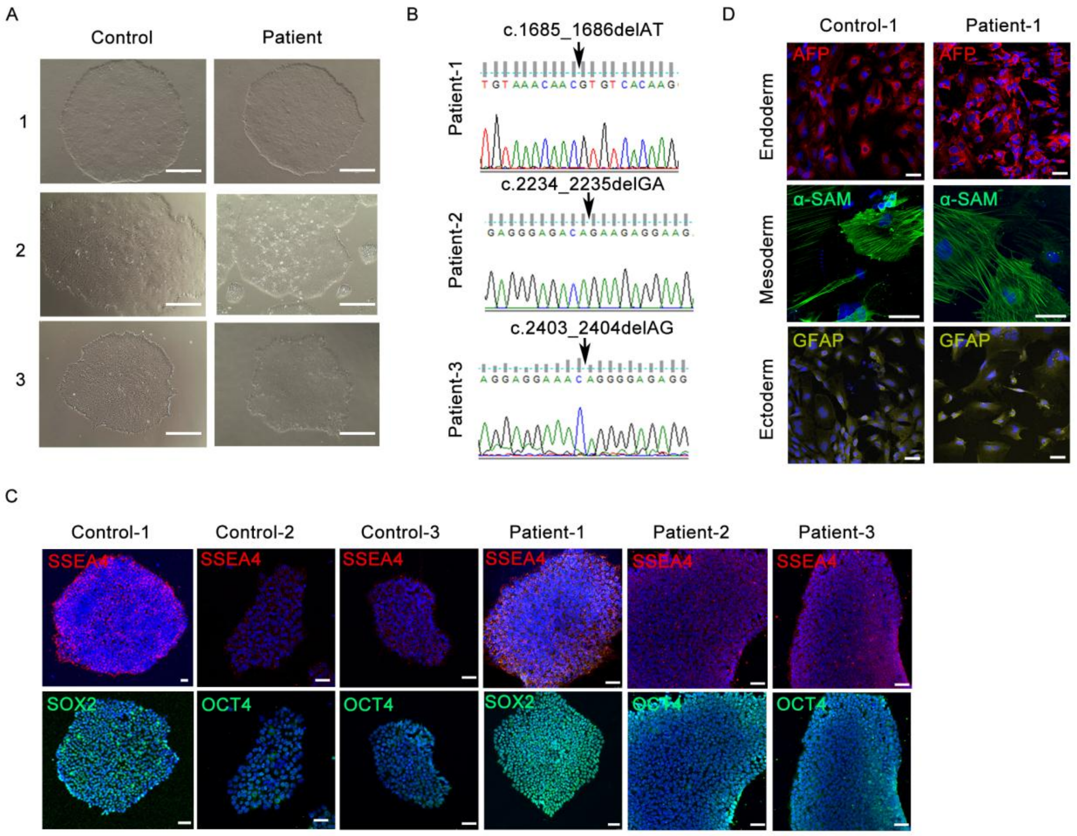

Figure S2. Generation and characterization of iPSCs from patients with RPGR mutations and healthy volunteers. Related to Figure 1 and Figure 3. (A) iPSC colonies are generated from three healthy volunteers and three patients and maintained in TeSR-E8 medium. Scale bar, 400 μm. (B) Identification of RPGR mutations in iPSCs via sequencing. (C) Immunostaining of the pluripotency markers SSEA4 (red), OCT4 (green) or SOX2 (green). Scale bar, 50 μm. (D) *In vitro* differentiation of normal and patient iPSCs. Immunostaining of marker proteins for endoderm (a-fetoprotein [AFP]), mesoderm (a-smooth muscle actin [a-SMA]) and ectoderm (GFAP) is shown. Scale bar, 50 μm.

Figure S3

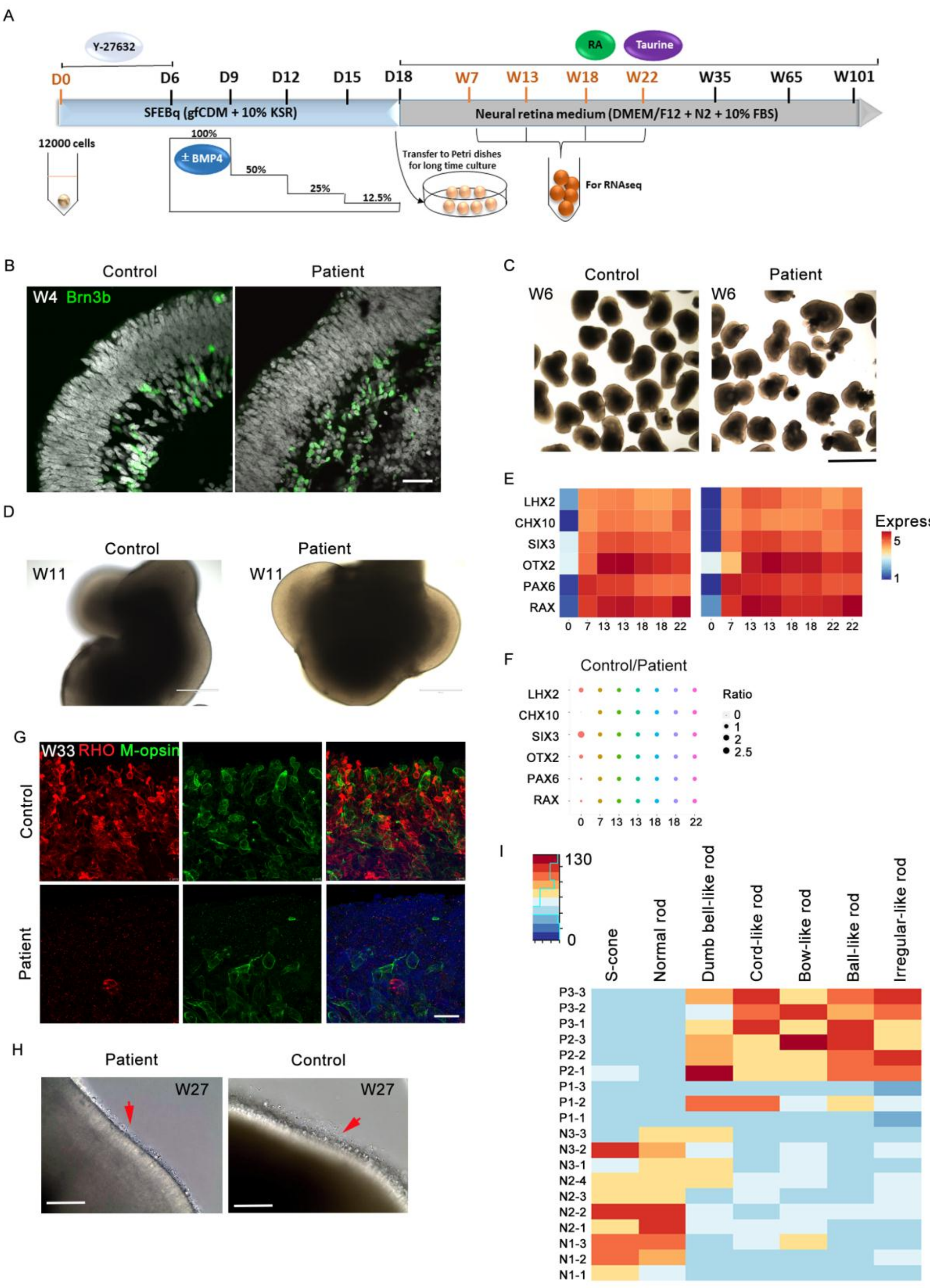

Figure S3. 3D Optic cups derived from patient and control iPSCs. Related to Figure 1 and Figure 3. (A) Schematic representation of differentiation timelines from iPSCs to 3D retinae based on the method previously described by Kuwahara et al. (2015). (B) Brn3b (green) is an early transcriptional marker of ganglion cells.

Retinal ganglion cells are located in the inner layer of the organoids. (C and D) Bright-field images of 3D retinal organoids derived from patient and normal iPSCs. Scale bars, 1 mm (C) and 400 μm (D). (E and F) According to RNAseq analysis, there are no obvious differences in eye field transcription of 3D retinal organoids derived from patient and control iPSCs. (G) Immunostaining of the rod marker Rhodopsin (red) and the L/M-cone marker L/M-opsin (green) showing significant differences in cell morphology and cell counts in retinal organoids at W33. Scale bar, 25 μm. (H) The outer segment-like structure (indicated with a red arrow) is much thicker in the normal optic cup than that in the patient one. Scale bar, 100 μm. (I) Heatmap showing the cell counts of blue cones and pathological and normal rods in the control (N) and patient (P) optic cups at W33. Numbers represent total cell counts in three random fields per sample. n=3 organoids for each cell type. Data are from three independent experiments.

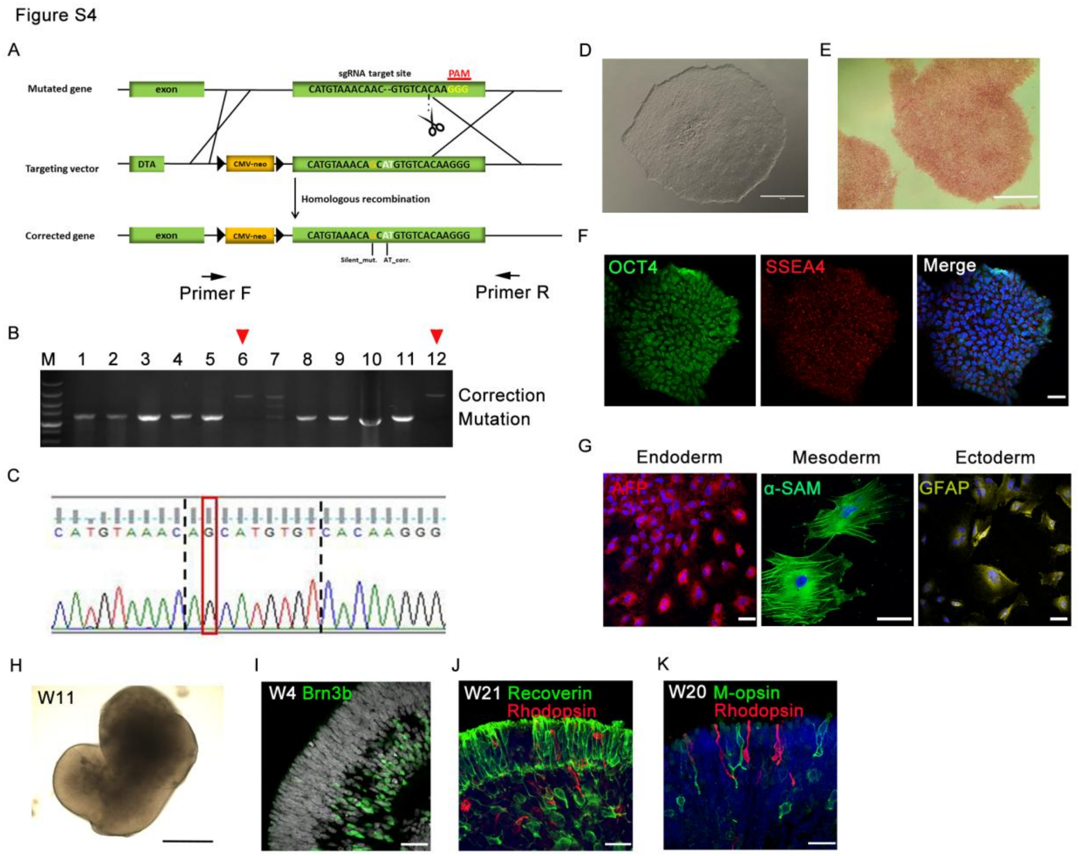

Figure S4. CRISPR/Cas9-mediated gene correction and 3D retinal differentiation of corrected iPSCs. Related to Figure 4. (A) Schematic representation of the targeted gene correction procedure using CRISPR/Cas9. (B) Corrected iPSCs were identified and isolated through amplification of single-cell clones and screening of the PCR products. (C) Corrected iPSC colonies were verified via sequencing. The red box indicates one silent mutation that was specifically introduced to distinguish from non-homologous end joining. (D) Corrected iPSC colonies were cultured in TeSR-E8 medium. Scale bar, 400 μm. (E) Corrected iPSC colonies express alkaline phosphatase. Scale bar, 400 μm. (F) Immunostaining of the pluripotency markers OCT4 (green) and SSEA4 (red). Scale bar, 50 μm. (G) *In vitro* differentiation of corrected iPSCs. Immunostaining of marker proteins representing

endoderm (a-fetoprotein [AFP]), mesoderm (a-smooth muscle actin [a-SMA]) and ectoderm (GFAP) is shown. Scale bar, 50 µm. (H) Bright-field image of 3D retinal organoids derived from corrected iPSCs. Scale bar, 400 µm. (I) The early transcriptional marker of ganglion cells Brn3b (green) can be observed at W4 in corrected retinal organoids. Scale bar, 20 µm. (J) Immunostaining of Recoverin (green) and the rod marker Rhodopisn (red) in corrected retinal cups at W21. Scale bar, 25 µm. (K) Immunostaining of the rod marker Rhodopsin (red) and the L/M-cone marker L/M-opsin (green) showing photoreceptors in W20 corrected retinal organoids aligned in the apical layer. Scale bar, 25 µm.

Figure S5

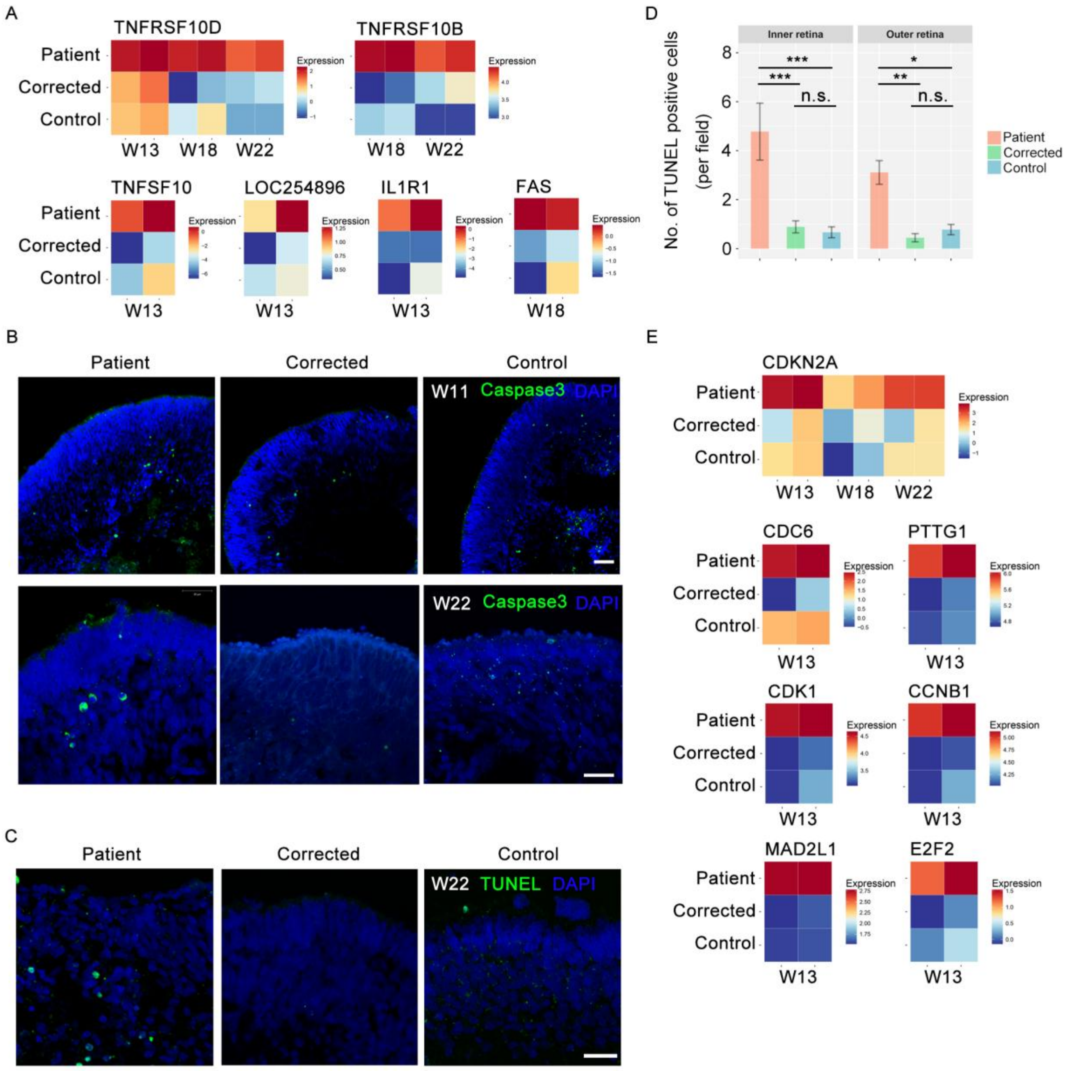

Figure S5. Detection of apoptosis and cell death in retinal organoids derived from iPSCs. Related to Figure 4. (A) Heatmaps showing the expression of genes in apoptotic signaling pathway. Two biological replicates were performed for the patient and corrected samples at W13, W18 and W22 and for the control samples at W13 and W18. Different colors represent the value of Log2 (FPKM+0.01), FDR<0.05. (B) Fluorescence images of active caspase 3 (green) staining in retinal organoids at W11 and W22. Scale bar, 20 µm. (C) TUNEL staining (green) in retinal organoids at W22. Scale bar 25 µm. (D) Quantification of TUNEL positive cell in (B). n=3 organoids for

each cell type. Data are from three independent experiments. (E) Heatmaps showing the expression of genes in cell cycle signaling pathway. Different colors represent the value of Log2 (FPKM+0.01), FDR<0.05.

Figure S6

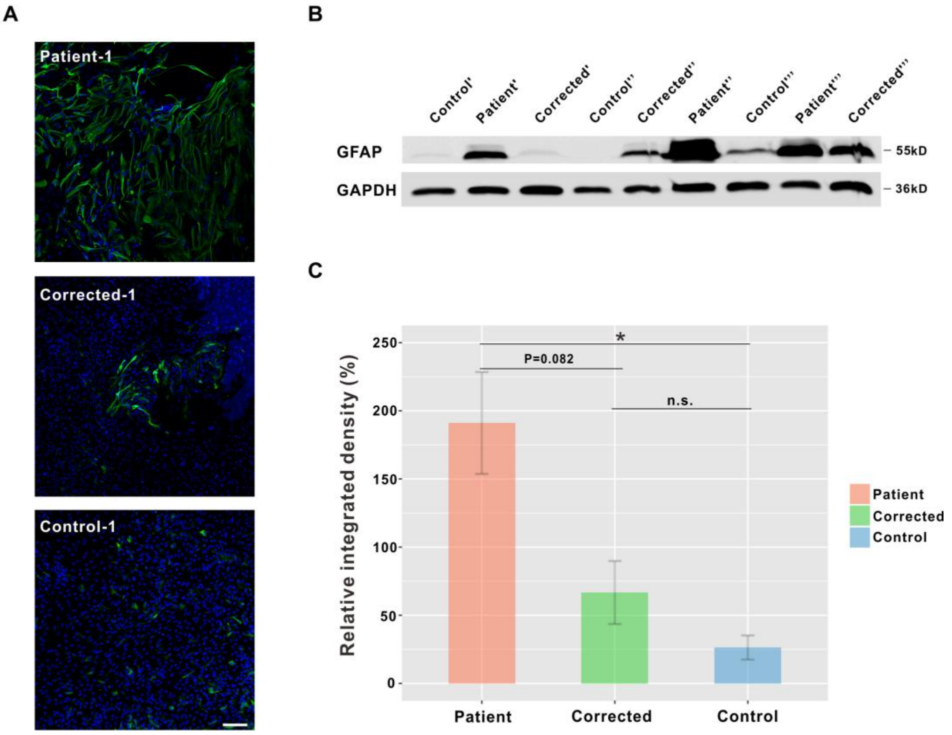

Figure S6. Up-regulated GFAP expression in patient retinal organoids. Related to Figure 4. (A) 3D retinal organoids were derived from patient-1, control-1 and corrected-1 at W75 and amplified in adhesive dishes for 7 weeks. The expression of GFAP in patient is much more than that in control and corrected ones. Scale bar, 100μm. (B) Three replicates of western blotting detection of GFAP in patient-1, control-1 and corrected-1 retinal organoids at W82, n=3 organoids for each cell type. (C) Quantitative analysis of GFAP protein in (B). Data are presented as mean value ± SEM of three independent experiments. Statistical significance was determined by * $p<0.05$ using unpaired *t* test.

Figure S7

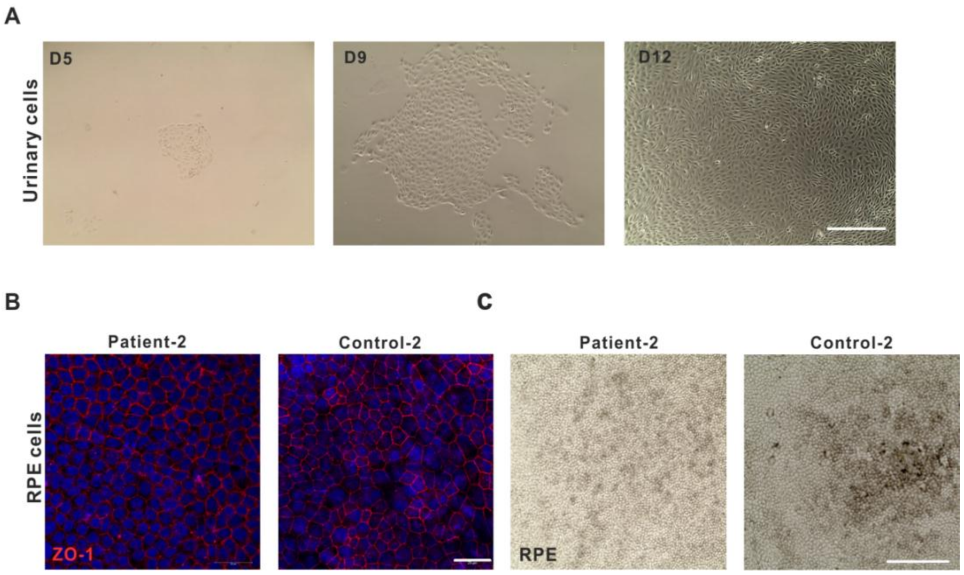

Figure S7. Morphology of urinary cells and iPSC-derived RPE cells. Related to Figure 7. (A) Urinary cells formed a small clone at day 5 and expanded slowly in 12 days. Scale bar, 400 μm. (B) Immunostaining of tight junction marker ZO-1(red) in patient-2 and control-2 iPSC-derived RPE cells after RPE induction for 60 days. Scale bar, 25 μm. (C) RPE cells showed polygon mesh and pigment appeared in part of RPE cell sheets at day 60. Scale bar, 200 μm.

Supplymental Tables

Table S1. Information of iPCSs derived from patients and controls

	Gender[a]	Age[b]	RPGR Mutation	Source	Reprogramming methods	Picked clone (used)[c]
**Patient-1**	M	24y	c.1685_1686 delAT	Urinary cells	Lentiviral pVSVG vector	3(2)
**Patient-2**	M	16y	c.2234_2235 delGA	Urinary cells	Cocktail plasmids (electrotransfection)	6(2)

**Patient-3**	M	18y	c.2403_2404 delAG	Urinary cells	Cocktail plasmids (electrotransfection)	2(2)
**Control-1**	M	4-5m (Fetal)		Fibroblast	Cocktail plasmids (electrotransfection)	3(2)
**Control-2**	F	24y		Urinary cells	Cocktail plasmids (electrotransfection)	6(2)
**Control-3**	M	49y		Urinary cells	Cocktail plasmids (electrotransfection)	3(2)
**Carrier-1**	F	49y	c.2403_2404 delAG	Urinary cells	Cocktail plasmids (electrotransfection)	3(2)

[a]Gender information. M, male; F, Female.

[b]Age of volunteers collecting fibrolast or urinary cells.

[c]No. of colony generated from primary cells and No. of colony used in this study are indicated in brackets.

Table S2. Compiled comparison of RNAseq data related to Figure 3 and 4 (Sheet 1), Figure 5 (Sheet 2), Figure 6 (Sheet 3), Figure S3 (Sheet 4), Figure S5 (Sheet 5) and other genes (Sheet 6). Supplied as a separate Excel file.

Extended Experimental Procedures

Patient Clinical Imaging

The macular pigment optical density (MPOD) examinations were used 1-wavelength-reflection and recorded reflection images (Visucam200, Carl Zeiss Meditec, Germany), so does the fundus images. Macula thickness OU: macular cube 512 x 128 and optical coherence tomography (OCT) scans (Cirrus HD-OCT, Carl Zeiss Meditec, Germany).

Isolation and Expansion of Urinary Cells

We isolated urinary cells from 100-300 ml of urine from three RPGR patients, one familial carrier and three controls as previously reported (Zhou et al., 2012). Urinary cells were cultured in primary medium containing DMEM and Ham's F12 nutrient mix (1:1), supplemented with 10% FBS (Biological Industries), 100 U ml^{-1} penicillin and 100 μg ml^{-1} streptomycin for 3 days. The primary medium was subsequently exchanged with proliferation medium containing REGM Bullet Kit (Lonza), DMEM supplemented with 10% FBS (Biological Industries), 1% GlutaMAX (Life Technologies), 1% NEAA (Sigma), 100 U ml^{-1} penicillin, 100 μg ml^{-1} streptomycin (Life Technologies), 5 ng ml^{-1} bFGF (Gibco), 5 ng ml^{-1} PDGF-AB (Gibco) and 5 ng ml^{-1} EGF (Gibco). Then, we changed half of the proliferation medium daily. Small colonies appeared within 3-5 days after plating and grew steadily. The cells were passaged after reaching 80-90% confluence.

Generation and Culture of iPSCs

RPGR patient-1 urinary cells were infected with viral supernatants generated via transfection of HEK293T cells (using Fugene, Promega) with a lentiviral pVSVG vector (Addgene) containing human Oct4, Sox2, Klf4, and c-Myc cDNAs. After 4 days, the infected cells were routinely trypsinized, and 50,000 of the cells were seeded into a 10-cm culture dish that had been coated with feeders one day earlier. The cells were cultured in human ESC medium that contained DMEM F12 nutrient mixture supplemented with 20% KSR (Gibco), 1% GlutaMAX (Life Technologies), 1% NEAA (Sigma), 100 U ml^{-1} penicillin, 100 μg ml^{-1} streptomycin (Life Technologies), 100 μM β-mercaptoethanol (Gibco) and 10 ng ml^{-1} bFGF (Gibco). After infection, the

medium was renewed daily at all stages. From day 16 onward, the colonies that were sufficiently large to identify as human ESC-like could be mechanically picked and expanded in TeSR™-E8™ (STEMCELL Technologies) medium on Matrigel (growth-factor-reduced; Corning). Normal control-1 iPSCs were generated from human fibroblasts using a cocktail of reprogramming plasmids encoding Oct4, Sox2, Lin28, Klf4, L-myc, p53shRNA, and the miR-302/367 cluster (Episomal iPSC Kit, System Biosciences) delivered by nucleofection using LONZA 4D. Patient-2,-3 and control-2,-3 iPSCs were generated from human urinary cells using the same cocktail of reprogramming plasmids above.

Retinal Organoid Differentiation from human iPSCs.

The procedure for inducing early stages of retinal organoid differentiation was based on a previously described protocol with some modifications (Kuwahara et al., 2015). Human iPSCs (patient-1, 2, control-1 and 2) were dissociated into single cells by TrypLE Select (Life Technologies) supplemented with 0.05 mg/ml DNase I (Roche) and 20 μM Y-27632 (Selleck). Single cells were reaggregated in low-cell-adhesion 96-well plates with V-bottomed conical wells (SumilonPrimeSurface plate; Sumitomo Bakelite) at a density of 12,000 cells per well in differentiation medium containing 10% KSR, 45% Iscove's modified Dulbecco's medium (IMDM, Gibco), 45% Hams F12 (F12, Gibco), Glutamax (Life Technologies), monothioglycerol (450 μM, Sigma), 100 U ml^{-1} penicillin and 100 μg ml^{-1} streptomycin (Gibco), supplemented with 20 μM Y-27632 (Sellock), under 5% CO_2 at 37 ℃. The day on which suspension culture was initiated was defined as day 0. On day 6, recombinant human BMP4 (R&D) was

added to the culture to a final concentration of 55 ng/ml, and its concentration was diluted in half every 3rd day via changing half of the medium.

On day 18, the aggregates were transferred into petri dish and cut into 2 to 3 pieces using a V-Lance Knife (Alcon Surgical). They were suspended in neural retina medium [DMEM/F12-Glutamax medium (Life Technologies) supplemented with 1% N-2 supplement (Life Technologies), 0.5 μM retinal acid (Sigma), 0.1 mM taurine (Sigma), 10% FBS (Biological Industries), 100 U ml^{-1} penicillin and 100 μg ml^{-1} streptomycin (Life Technologies)] under 5% CO_2 conditions for long term culture. The medium was changed every 7th day protecting from light exposure, especially after 120 days.

RPE cells differentiation

Human iPSCs were cultured for 3 to 4 days to get nearly 80% confluency. They were treated with 10μM Y-27632 (Selleck) for 1h before digested with 0.5mM EDTA (Gibco) 5-8 minutes. Approximately $8.8X10^2$ aggregates per 1ml were cultured in suspension for three days in medium 1, which contained 80% DMEM/F12 (Gibco), 0.1mM 2-Mercaptoethanol (Gibco), 0.1mM Non-essential amino acid (Sigma), 2mM L-glutamine (Gibco), 20% KSR (Gibco), 5μM CKI-7 (Sigma), 5μM SB431542 (Sigma) and 10μM Y-27632. On day 4, those aggregates were transferred to medium 2 containing 80% GMEM (Gibco), 0.1mM 2-Mercaptoethanol, 0.1mM Non-essential amino acid, 1mM Sodium Pyruvate (Sigma), 20% KSR, 5μM CKI-7, 5μM SB431542 and 10μM Y-27632. After 3 days, medium 3 should be used for another 9 days, which included 80% GMEM, 0.1mM 2-Mercaptoethanol ,0.1mM Non-essential amino acid,

1mM Pyruvate, 1% Pencilin-Streptomycin liquid (Gibco), 15% KSR, 5μM CKI-7, 5μM SB431542 and 10μM Y-27632. The medium 3 would be changed every three days. Then changed medium 4 containing 80% GMEM, 0.1mM 2-Mercaptoethanol, 0.1mM Non-essential amino acid, 1mM Pyruvate, 1% PS, 10% KSR, 5μM CKI-7 and 5μM SB431542 every 3rd day for 6 days. On day 21, those aggregates were plated on Matrigel-coated dishes in small molecules removed medium 5, containing 80% GMEM, 0.1mM 2-Mercaptoethanol, 0.1mM Non-essential amino acid, 1mM Pyruvate, 1% PS and 10% KSR. The medium was changed every 3rd day. On day 40, the polygon mesh cells were digested into single cells with 0.25% Trypsin-EDTA (Life technology) for 8 minutes. Single cells were then cultured on Matrigel-coated dishes in medium 5. RPE cells would cover all the dishes within 5-7 days and then maintained in RPEM medium containing 97% DMEM/F12, 2% B27 (Gibco) and 1% PS. After two weeks, pigment would form in most of the polygon mesh RPE cells. After 72h serum starving, RPE cells derived from patient-1, -2, -3, control-1, -2, -3 and corrected-1 iPSCs would be used for immunostaining of typical RPE markers and cilia markers.

Alkaline Phosphatase Staining

Human iPSCs were fixed with 4% PFA for 10 min, then rinsed with TBST buffer (20 mM Tris-HCl, pH 7.4, 150 mM NaCl, 0.1% Tween-20) and stained with Naphthol/Fast Red TR Solution [mixture of 25 mM Tris-Cl (pH 9.0), 0.4 mg/ml α-naphthyl phosphate (Sigma), 1 mg/ml Fast Red TR (Sigma), 8 mM $MgCl_2$] for 15-30 min in the dark. The cells were then rinsed with TBST and imaged under

microscope.

Embryonic Body (EB) Formation and *in vitro* Differentiation

Human iPSCs on a Matrigel-coated plate were dissociated with 0.5 mM EDTA and resuspended in EB medium containing DMEM/F12 (Gibco), 20% Knockout serum replacement, KSR (Gibco), 1% L-GlutaMax (Life Technologies), 1% NEAA (Sigma), and 0.1mM β-mercaptoethanol (Gibco). The cells were plated in suspension in ultra-low attachment culture dishes (Nunc) for 8 days and then transferred to a Matrigel-coated plate and cultured for an additional 16 days. The cells were then collected and analyzed for markers of three germ tissue lineages: ectoderm (GFAP), mesoderm (α-SMA), and endoderm (AFP). Each marker was detected via immunofluorescence.

Sectioning of hiPSC-Derived 3D Retinae with a Vibratome

3D retinae were fixed in 4% paraformaldehyde for 60 min at 4 ℃ before embedding in 2% agarose in PBS. Sections of 50 μm were prepared using a vibratome (VT1000S, Leica) and subjected to immunohistochemistry immediately or kept at 4 ℃.

Immunofluorescence

Cells growing on slides were fixed in 4% paraformaldehyde at room temperature for 15 minutes and then transferred to 4 ℃ for 60 minutes. Cells, cryosection or vibratome slide sections rinsed in PBS, and then incubated in blocking buffer (4% bovine serum albumin and 0.5% Triton X-100 in PBS) for 1 hour at room temperature, prior to incubation with the primary antibodies overnight at 4 ℃. After secondary labeling, nuclei were visualized using DAPI (2 μg/ml) staining in all imaged samples.

RNA Extraction and RNA Sequencing

Total RNA was extracted from retinal organoids using the RNeasy Mini Kit (Qiagen) and TRIzol reagent (Life Technologies) according to the manufacturer's protocols. A total of 120 µg of RNA from the patient, control and corrected 3-D retinae from a series differentiation times (W0, 7, 13, 18 and 22) was used for Illumina library preparation (Biomarker Technologies, Beijing, China). Significant was determined by adjusted *p* value <0.05.

Quantification of photoreceptor cells

According to the stratified 3D retinae, a clear line can be drawn between the outer retina (including outer nuclear layer, inner and outer segments) and the inner retina (containing plexiform layers and ganglion cells layer). The photoreceptor cell markers, Rhodopsin, L/M-opsin, S-opsin and Recoverin were mainly expressed in the outer retina, whilst a few located in the inner retina. The percentage of Recoverin positive cells was calculated by the analysis system in Leica TCS SP8 confocal microscope. For other opsin positive cells, same parameters (Z-stack, sections, pixel, fluorescence intensity, etc.) were used in each picture. The average cell number of nine random fields (63X magnification) from three samples was calculated by counting manually.

KEY RESOURCES TABLE

REAGENT OR RESOURCE	SOURCE	DENTIFIER
Antibodies		
Actin, α-Smooth Muscle antibody	Sigma	Cat# A5228
AFP	R&D Systems	Cat# MAB1368-SP
SSEA4	Abcam	Cat#AB16287
GFAP	Life Technologies	Cat#13-0300
Oct4	Abcam	Cat#AB80893

Sox2	Santa Cruz Biotechnology	Cat# sc-17319
Rhodopsin	Sigma	Cat# O4886
Brn3b	Santa Cruz Biotechnology	Cat# sc-6026
Recoverin	Millipore	Cat# AB5585
GT335	AdipoGen	Cat# AG-20B-0020
Arl13b	ProteinTech	Cat# 17711-1-ap
PKCα	Sigma	Cat# P4334
L/M-opsin	Millipore	Cat# AB5405
S-opsin	Millipore	Cat# AB5407
VGLUT 1	Millipore	Cat#AB5905
Synaptophysin	Abcam	Cat#AB32127
ZO-1	Invitrogen	Cat#339100
HCN1	Abcam	Cat#AB176304
Chemicals, Peptides, and Recombinant Proteins		
GlutaMAX Supplement	Life Technologies	Cat# 35050-061
Non-essential Amino Acid Solution (100×)	Sigma	Cat# M7145
bFGF Recombinant Human Protein	Gibco	Cat# 13256029
PDGF-AB Recombinant Human Protein	Gibco	Cat# PHG0134
EGF Recombinant Human Protein	Gibco	Cat# PHG0311
Fugene	Promega	Cat# E2311
KnockOut™ Serum Replacement	Gibco	Cat# A3181502
β-mercaptoethanol	Gibco	Cat# 21985-023
TeSR™-E8™	STEMCELL Technologies	Cat# 05940
Matrigel, Growth Factor Reduced (GFR) Basement Membrane Matrix, Phenol Red-Free, *LDEV-Free	Corning	Cat# 356231
Y-27632	Selleck	Cat# S1049
Monothioglycerol	Sigma	Cat# 96-27-5
Recombinant human BMP4	R&D Systems	Cat# 314-BP
N-2 Supplement (100X)	Life Technologies	Cat# 17502-048
V-bottomed conical wells plate	SumilonPrimeSurface Sumitomo Bakelite	Cat# MS-9096V
CKI-7 dihydrochloride	Sigma	Cat# C0742-5MG
SB-431542 hydrate	Sigma	Cat# S4317-5MG
B-27® Supplement (50X)	Gibco	Cat# 0080085-SA

GMEM	Gibcao	Cat# 11710-035
Critical Commercial Assays		
REGM Bullet Kit	Lonza	Cat# CC-3190
P3 Primary Cell 4D-Nucleofector™ Kit	Lonza	Cat# V4XP-3024
Episomal iPSC Kit	System Biosciences	Cat# SC900A-1
RNeasy Mini Kit	Qiagen	Cat# 74104
Oligonucleotides		
pX330-sgRNA-F	CACCGCATGTAAACAACGTGTCACAA	
pX330-sgRNA-R	AAACTTGTGACACGTTGTTTACATGC	
Primer F for correction verification	CACAGACTAGAGAGTGGCAC	
Primer R for correction verification	CCTCTACCCTTGTCTTTCTC	
pX330	Addgene	Plasmid #42230
Software and Algorithms		
GraphPad Prism	GraphPad Software Inc	http://www.graphpad.com/scientific-software/prism/
Leica software	Leica	http://www.leica-microsystems.com/home/

Movies

Movie S1. Related to Figure 1. Differentiated rod (Rhodopsin, red) and S-cone (S-opsin, green) showing the outer segment-like structure (a higher magnification view).

Movie S2. Related to Figure 1. A lower magnification view of a differentiated 3D organoid showing the distribution of rods (Rhodopsin, red) and L/M cones (M-opsin, green).

Supplemental references

Kuwahara, A., Ozone, C., Nakano, T., Saito, K., Eiraku, M., and Sasai, Y. (2015). Generation of a ciliary margin-like stem cell niche from self-organizing human retinal

tissue. Nature Communications *6*, 6286.

Zhou, T., Benda, C., Dunzinger, S., Huang, Y., Ho, J.C., Yang, J., Wang, Y., Zhang, Y., Zhuang, Q., Li, Y., *et al.* (2012). Generation of human induced pluripotent stem cells from urine samples. Nature Protocols *7*, 2080-2089.
